# Single-shot isotropic differential interference contrast microscopy

**DOI:** 10.1038/s41467-023-37606-6

**Published:** 2023-04-12

**Authors:** Xinwei Wang, Hao Wang, Jinlu Wang, Xingsi Liu, Huijie Hao, You Sin Tan, Yilei Zhang, He Zhang, Xiangyan Ding, Weisong Zhao, Yuhang Wang, Zhengang Lu, Jian Liu, Joel K. W. Yang, Jiubin Tan, Haoyu Li, Cheng-Wei Qiu, Guangwei Hu, Xumin Ding

**Affiliations:** 1 Advanced Microscopy and Instrumentation Research Center, School of Instrumentation Science and Engineering, Harbin Institute of Technology, Harbin, 150080 China; 2 School of Electrical and Electronic Engineering, 50 Nanyang Avenue, Nanyang Technological University, Singapore, 639798 Singapore; 3 Engineering Product Development, Singapore University of Technology and Design, Singapore, 487372 Singapore; 4 Department of Medical Oncology, Harbin Medical University Cancer Hospital, Harbin Medical University, Harbin, 150081 Heilongjiang China; 5 Department of Electrical and Computer Engineering, National University of Singapore, Singapore, 117583 Singapore; 6 Center of Ultra-Precision Optoelectronic Instrument engineering, Harbin Institute of Technology, Harbin, 150080 China; 7 Key Lab of Ultra-Precision Intelligent Instrumentation (Harbin Institute of Technology), Ministry of Industry and Information Technology, Harbin, 150080 China; 8 College of Mechanical and Electrical engineering, Northeast Forestry University, Harbin, 150040 Heilongjiang China; 9 Institute of Materials Research and Engineering (IMRE), A*STAR (Agency for Science, Technology and Research), 2 Fusionopolis Way, Singapore, 138634 Singapore

**Keywords:** Interference microscopy, Nanophotonics and plasmonics, Metamaterials

## Abstract

Differential interference contrast (DIC) microscopy allows high-contrast, low-phototoxicity, and label-free imaging of transparent biological objects, and has been applied in the field of cellular morphology, cell segmentation, particle tracking, optical measurement and others. Commercial DIC microscopy based on Nomarski or Wollaston prism resorts to the interference of two polarized waves with a lateral differential offset (shear) and axial phase shift (bias). However, the shear generated by these prisms is limited to the rectilinear direction, unfortunately resulting in anisotropic contrast imaging. Here we propose an ultracompact metasurface-assisted isotropic DIC (i-DIC) microscopy based on a grand original pattern of radial shear interferometry, that converts the rectilinear shear into rotationally symmetric along radial direction, enabling single-shot isotropic imaging capabilities. The i-DIC presents a complementary fusion of typical meta-optics, traditional microscopes and integrated optical system, and showcases the promising and synergetic advancements in edge detection, particle motion tracking, and label-free cellular imaging.

## Abstract (web summary)

The authors present a metasurface-assisted isotropic DIC microscopy technique. It is based on an original pattern of radial shear interferometry, that converts rectilinear shear into rotationally symmetric radial shear, enabling single-shot isotropic imaging capabilities.

## Introduction

Conventional wide-field microscopy primarily relies on optical absorption difference, but suffers from low contrast for transparent bio-specimens which have weak optical absorptivity^1^. Staining and fluorescent labels are commonly exploited to overcome this problem^2^. However, dyes are usually toxic to living cells and would affect cell viability while auxiliary labels^3,4^ suffer from photobleaching and phototoxicity^5^. Alternatively, label-free microscopy can mitigate those issues via encoding phase information from the refractive index difference of bio-samples into intensity distribution^6,7^. Particularly, DIC microscopy can record phase variations induced by the lateral shear between two orthogonally polarized lights to recover fine details, featuring the advantages of high contrast, fine spatial resolution and pseudo-3D relief imaging and free from the halo and shade-off artifacts of phase contrast (PC) microscopy^8^. Hence, it is widely used in measuring cellular morphology and segmentation, particle tracking and other applications^9^. However, conventional or so-called anisotropic DIC (a-DIC) microscopy^8^ has strong orientation sensitivity as it relies on Normarski prisms to generate the rectilinear shear between two orthogonal linear polarizations (LPs). Hence, the image contrast is created along that direction, but absent in the orthogonal. The lost information can be gained via mechanically rotating the specimen or prism^10,11^, but the single-frame image is still anisotropic, which is unsuitable for fast imaging of live specimens. So far, obtaining a single-shot i-DIC microscopy for fast phase imaging remains a critical challenge, due to the limited functionalities of conventional optical elements.

In parallel, metasurfaces are widely investigated for analog information processing and imaging applications thanks to their unprecedented capabilities of controlling light manipulations at subwavelength scale^12–15^. For instance, real-time edge detection is demonstrated with metasurfaces that can perform first-order^16–19^ and second-order (or Laplacian)^20–22^ analog differentiations in the frequency domain based on 4*f* or 2*f* metamaterial systems. Moreover, this can be extended into spatial domains^23–28^, where metasurfaces render scattering characteristics dependent on the linear momentum and function like the photonic crystal^29–34^ and multilayer thin film^35–37^ but with a much thinner profile. However, most of those implementations require opaque samples as they deal with amplitude signals. An exception is that ref. ^20^ demonstrates a two-dimensional optical spatial differentiation of amplitude and phase signals, which is based on bulky 4*f* schemes. For purely phase information enhancement, metasurface-based PC microscopy utilized a spiral phase to perform a two-dimensional spatial differentiation and isotropic edge detection in 4*f* system^38^, which can be enhanced via the further incorporation of hyperbolic phase for compressed imaging^39^. Nevertheless, they are PC imaging, which depends on the interference between incident and scattered fields and suffers from the halo and shade-off artifacts^8^. Recently, the miniaturized quantitative phase gradient microscope (QPGM) is proposed based on bilayer metasurfaces^40^, which however requires complex fabrication and sophisticated alignments. Such qualitative phase imaging has also been realized in a Fourier optical spin splitting microscopy (FOSSM)^41^ or metasurface-based transport of intensity^42^, all of which work in 4*f* system and are bounded to anisotropic imaging.

**Fig. 1:**
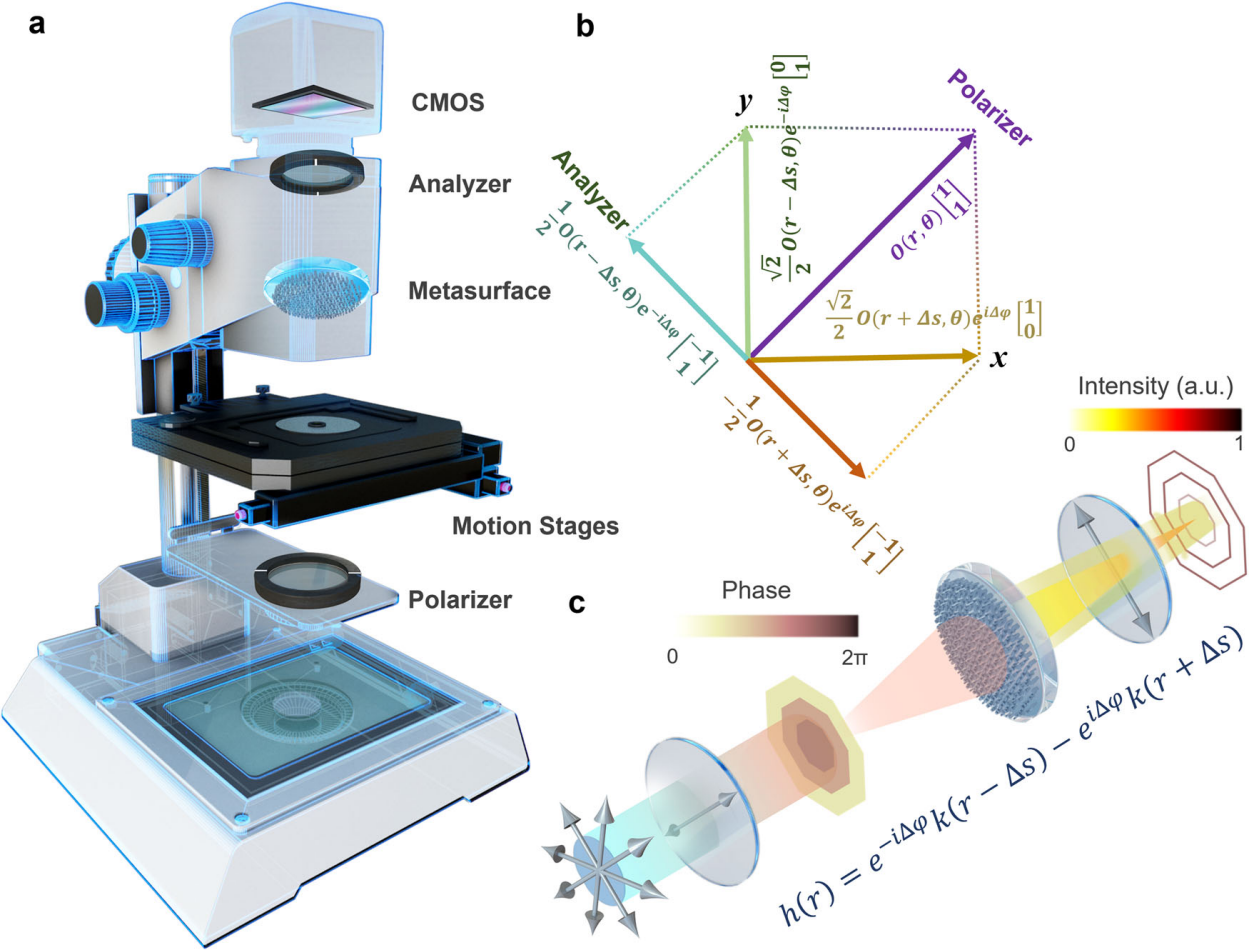
Principle of metasurface-assisted i-DIC microscopy. **a** The schematic of the proposed i-DIC microscopy. CMOS, complementary metal oxide semiconductor. **b** the polarization conversion and optical field changes in the polarization coordinate system of i-DIC microscopy. **c** The optical setup of i-DIC microscopy, The silver-white arrows indicate the polarization direction, and the function represents the APSF of i-DIC microscopy. a. u. arbitrary units.

In this paper, we theoretically propose and experimentally demonstrate a single-shot isotropic DIC (i-DIC) microscopy in a 2*f* system based on a single-layer metasurface (Fig. 1). We exploit the nontrivial independent phase modulation of two orthogonal polarization states^43–45^ to apply the desired rotationally symmetric shear along radial directions in polar instead of Cartesian coordinate, thus breaking orientation sensitivity of conventional DIC microscopy. To showcase the unique capability of metasurface-assisted i-DIC, we first realize the direct real-time tracking of microparticles, even though there are strong background scatterings. This would be important in studying dynamics of biomolecules in complex cellular environments for targeted drug delivery and other applications. Furthermore, our i-DIC microscopy is employed to capture breast cancer cells and tissues and demonstrate superior performance with much sharper images, which fuses meta-optics with cancer screening and diagnosis and other bio-applications. Detailed scrutiny of the state-of-the-art, as shown in Table 1, shows the impressive performance of our single-shot, isotropic, highly flexible, phase-based optical imaging capability using only a single metasurface. Our work unveils numerous opportunities in high-contrast biological imaging, particle tracking and other technologies, which may serve as an add-on compact module to be easily integrated in the existing imaging system.

**Table 1:** Comparison of commercial DIC, metasurface-based optical analog computing, optical imaging, and our i-DIC system

Classification	Ref.	Isotropic (Yes or No)	Amplitude- or phase-based imaging	Optical system	Principle
Commercial DIC	–	×	Amplitude and phase	Lens and birefringent prism	DIC imaging
Optical analog computing	Ref. ^16,19^	√	Amplitude	4*f*^a^	Frequency-domain filtering (Fourier transform)
	Ref. ^17,18^	×	Amplitude	4*f*	
	Ref. ^20^	√	Amplitude and phase	4*f*	
	Ref. ^21,22^	√	Amplitude	2*f*^b^	
	Ref. ^25–33^	√	Amplitude	Single layer	Spatial-domain filtering (Laplace transform)
	Ref. ^23,24,34^	×	Amplitude	Single layer	
	Ref. ^35–37^	√	Amplitude	Multilayer thin film	
Optical imaging	Ref. ^38^	√	Amplitude and phase	4*f*	PC imaging
	Ref. ^39^	√	Amplitude and phase	Single layer(2*f*)	
	Ref. ^40^	×	Amplitude and phase	Double layers	QPGM imaging
	Ref. ^41^	×	Amplitude and phase	4*f*	FOSSM imaging
	Ref. ^42^	-	Phase	4*f*	MS-TIE
★This work		√	Amplitude and phase	Single layer(2*f*)	i-DIC imaging

^a^The 4*f* system is a double-lens optical imaging or relay system with the input plane located one focal length (*f*_1_) in front of Lens 1 and the output plane located one focal length (*f*_2_) after Lens 2.

^b^The 2*f* system is a single-lens optical imaging system with the location of input plane *a* and the output plane *b* satisfied the Gaussian imaging Eq. (1)/*a* + 1/*b* = 1/*f*.

## Results

### Fundamental mechanisms of metasurface-assisted i-DIC microscopy

DIC microscope images are generated from the interference of two orthogonal polarized light beams that have spatial offset at the object plane, due to index contrast of the specimen and its surrounding, thus converting the phase gradient into amplitude signals. In conventional DIC microscopy, the sample is illuminated by a plane wave with LP angle at 45° and the corresponding complex amplitude transmission function *O*(*x*, *y*) can be decomposed into two LP components along *x* and *y* direction with equal amplitudes, as shown in Supplementary Note 1. Such two orthogonal LP components are modulated via the Normarski prisms with the encoding of phase by shear (2Δ*s*) and bias (2Δ*φ*) along the propagation. The objective and tube lens constitute the microscopic imaging system, and the imaging results are given by $O(x\pm \Delta s,y){e}^{\pm i\Delta \varphi }$, where positive and negative signs correspond to *x-* and *y-*polarizations, respectively. Those two images are re-combined by a linear analyzer at −45°, forming the phase difference contrast for imaging only at the direction perpendicular to the shear. Its interference pattern can be written as

1 $${I}_{{aniso}}\propto {{{{{{\rm{|}}}}}}O\left(x-\Delta s,y\right){e}^{-i\Delta \varphi }-O(x+\Delta s,y){e}^{i\Delta \varphi }{{{{{\rm{|}}}}}}}^{2}$$

This conventional DIC microscopy can also be achieved via a judiciously designed metasurface, and the corresponding theory and design can be found in Supplementary Note 2, where the shear is asymmetric. Consequently, such a system is still anisotropic, necessitating the second-frame imaging or adjustment of optical elements to break the asymmetric limitation for the recovery of lost information in the other direction^11,12^.

To reveal the full information in a single shot, we resort to an isotropic shear in the radial direction of a polar coordinate, which is rotationally symmetric, as illustrated in Fig. 1. The output signals can be described as $O(r\pm \Delta s){e}^{\pm i\Delta \varphi }$, where positive and negative signs represent two orthogonal LP states. The final output image after the analyzer would be

2 $${I}_{{iso}}\propto {{{{{{\rm{|}}}}}}O\left(r-\Delta s\right){e}^{-i\Delta \varphi }-O(r+\Delta s){e}^{i\Delta \varphi }{{{{{\rm{|}}}}}}}^{2}$$

which forms the i-DIC microscopy and captures the information along all the directions simultaneously. In the optical setup, a plane wave passes through polarizer 1 and generates a monochromatic polarized beam with a polarization angle of 45° to illuminate the sample. The analyzer makes two orthogonally polarized waves to be projected in the direction of −45° to produce the polarization-encoded interference image captured by CMOS camera. Importantly, such a modulation can overlap two polarized images in the image plane along radial directions, thus enabling the isotropic contrast in our i-DIC microscopy.

Without loss of generality, for our i-DIC microscopy with finite optical pupil and optical aberration, the coherent image system can be described by the amplitude point spread function (APSF). The APSF of widefield microscopy is denoted as *k*(*r*), and thus the APSF of i-DIC can be described as

3 $$h\left(r\right)={e}^{-i\Delta \varphi }k\left(r-\Delta s\right)-{e}^{i\Delta \varphi }k\left(r+\Delta s\right)$$

The design principle and detailed description of metasurface-assisted DIC microscopy can be found in Supplementary Note 2 and 3. For a phase-only target with unity amplitude (undetectable under the conventional wide-field microscopy), the phase variance and edge information along every direction can be preserved in our i-DIC microscopy, as shown in Supplementary Note 4. Beyond the common advantages of phase sensitive microscopy such as label-free and non-toxicity features, the phase imaging of i-DIC with radial shear we realized here breaks the limitation of traditional optical elements bounded by LP response, which reflects the unique and exclusive advantages of our imaging system. For all types of samples including rectangular or square objects with sharp right-angles corners, our i-DIC microscopy does not only get the edge information of all directions, but also can obtain additional performance, e.g. corner detection, as shown in Supplementary Note 5. Moreover, our single-layer metasurface operates as a 2*f* system and its multifunctionality and ultrathin thickness can further allow the miniaturization and integration of microscopy.

### Implementation of metasurface-assisted DIC microscopy

**Fig. 2:**
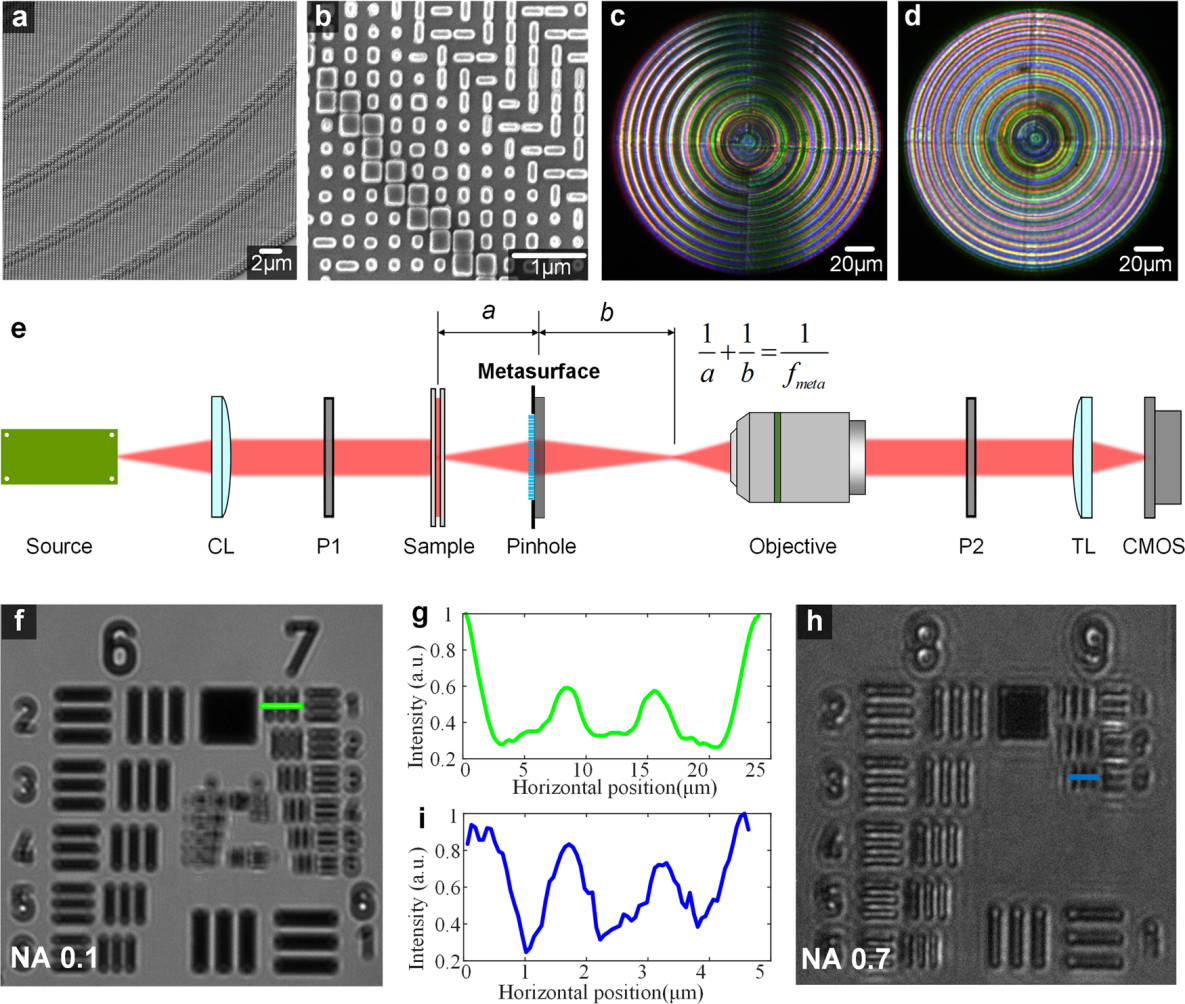
Characterization and performance tests of the metasurfaces. **a**, **b** SEM image of the fabricated metasurface. Images of a-DIC (**c**) microscopy and i-DIC (**d**) microscopy under white-light illumination in transmission mode. **e** The schematic configuration of the metasurface DIC microscopy. CL, collimating lens, P1 and P2, a pair of crossed polarizers. TL, tube lens. CMOS complementary metal oxide semiconductor. **f**–**i** Imaging result of the 1951 USAF test chart and Horizontal cross-section of the intensity distributions with NA 0.1 (**f**, **g**) and NA 0.7 (**h**, **i**). a. u. arbitrary units. The designed parameters of metasurfaces used in panel **f** (panel **h**) are the diameter D = 200 μm (200 μm), focal length *f* = 1 mm (100 μm), shear 2Δs = 1 μm (0.4 μm), and the bias 2Δφ = π (π).

To compare the performance of a-DIC and i-DIC microscopy, we designed, fabricated, and further tested the metasurfaces at the wavelength of 620 nm (see Methods for the fabrication details). The elaborated metasurface is based on polarization multiplexing principle and can achieve three abilities simultaneously, i.e. optical imaging (focusing), separation of polarized beams (shear) and the axial phase-shift (bias). The selected unit cells to satisfy independent phase control under two cross polarizations can be found in Supplementary Note 6, and the meta-atoms structure can be seen from the scanning electron microscopy (SEM) images in Fig. 2a, b. The focal properties of the demonstrated DIC metasurface are characterized using the optical setup in Supplementary Note 7, and the simulated and measured focal spots in a-DIC and i-DIC microscopes are shown in Supplementary Note 8. For a-DIC microscopy, the focal spot is elliptic while it’s circular for i-DIC microscopy. The sizes and shapes of the measured focal spots are in good agreement with the simulation, which verifies the theoretical transfer function and APSF of the a-DIC and i-DIC metasurface imaging system. The a-DIC and i-DIC metasurfaces under white-light illumination at transmission mode are comparatively shown in Fig. 2c, d, for which a pair of crossed polarizers are placed on both sides. Despite the presence of chromatic aberration, the a-DIC metasurface shows strong directional dependence while the i-DIC one is rotationally symmetric and has no directivity.

We illustrate the experimental setup of metasurface-assisted a-DIC and i-DIC microscopy in Fig. 2e. The monochromatic polarized light is obtained from a red LED light source at the wavelength of 620 nm via a collimator and a polarizer. The metasurface is used to image the sample, with a pinhole (diameter of 200 µm) placed just in front of it to block the background light and external noise. Polarizers 1 and 2 are placed along ±45°, respectively. It is worth noting we here adopt the redundant object lens and tube lens as the secondary imaging system to transfer the image plane of the metasurface to CMOS camera, for the sake of convenience of experimental test in the lab. Nevertheless, they are not the essential module of our designed i-DIC and can be removed in practice. In general, our 2*f* i-DIC imaging system can be more compact via directly mounting a single-layer metasurface and a pair of polarizers on the camera sensor^46,47^.

The resolution of DIC microscopy is consistent with wide-field microscopy, which is still limited by numerical aperture (NA) and wavelength. We experimentally demonstrate the edge-detection performance and quantify the resolution of the metasurface-assisted DIC microscopy with United States Air Force 1951 (USAF 1951) resolution test chart. The group 7 element 1 (3.906 μm in linewidth) can be resolved from the imaging result and cross-section for the DIC metasurface with NA = 0.1 (see Fig. 2f, g), while the group 9 element 3 with 0.775 μm in linewidth can be resolved by a DIC metasurface with NA = 0.7 (see Fig. 2h, i). The results verify the diffraction-limited resolution of our microscopy conforms to the intended design (3.782 μm for NA 0.1, 0.54 μm for NA 0.7 on the basis of Rayleigh Criterion). It is worth mentioning that DIC microscopy is developed to tackle the issue of phase visualization, while the DIC microscope can also perform edge detection for amplitude samples^8^. The detailed description can be seen in Supplementary Note 9.

### Comparison between metasurface-assisted a-DIC and i-DIC microscopy

**Fig. 3:**
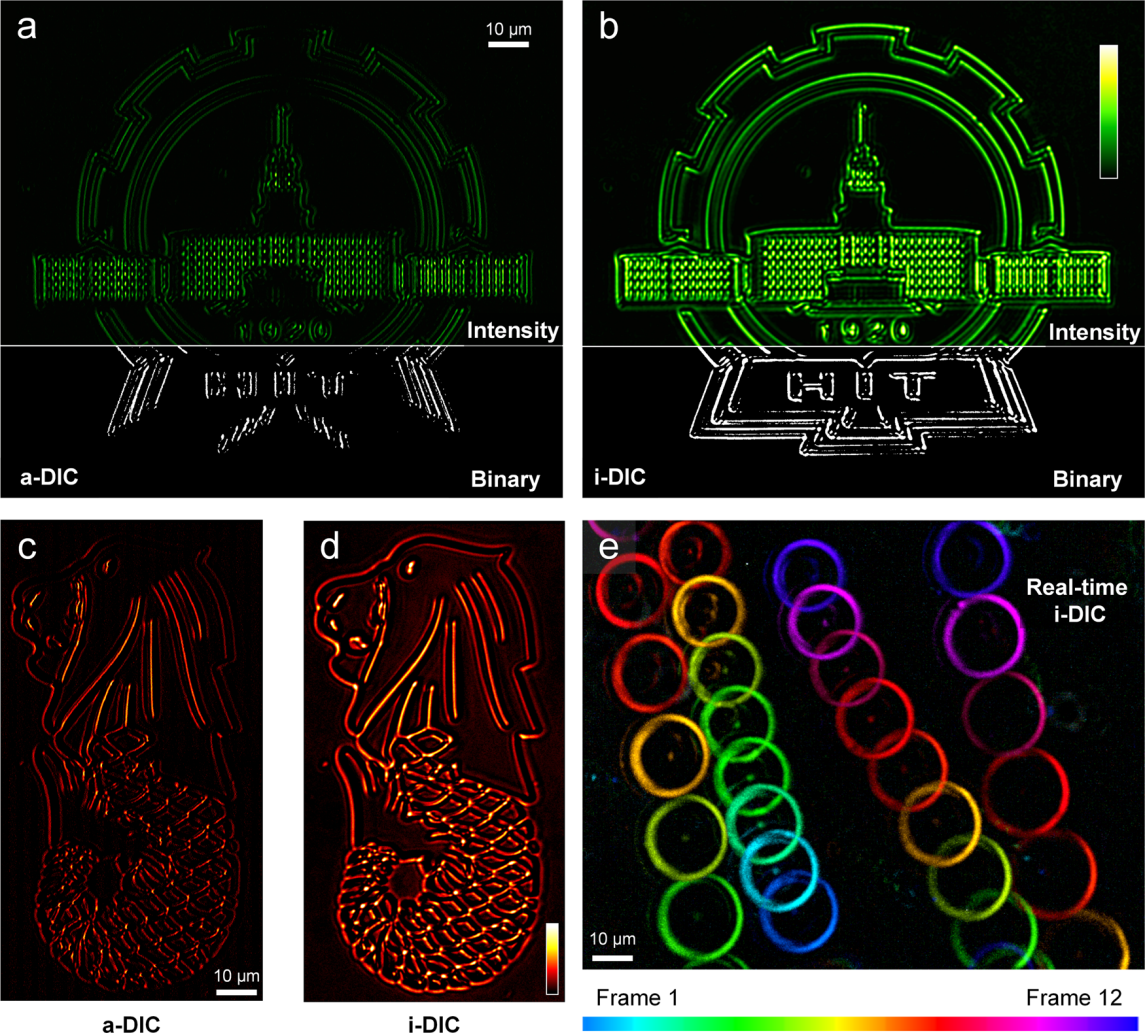
Imaging results with a-DIC and i-DIC microscopy. The high-resolution icon imaging results in a-DIC microscopy (**a**, **c**), i-DIC microscopy (**b**, **d**) with NA = 0.7, λ = 620 nm. **e** The motion tracking of SiO_2_ microspheres.

We first characterize the capability of enhanced edge detection, an important function with reduced date volume in locating and identifying the targets for data compression, object inspection, microscopy and general computer vision, of our metasurface-assisted DIC imaging of transparent samples. For experimental demonstrations, we use the metasurface with a high NA (NA = 0.7, see Fig. 2d). The a-DIC and i-DIC results of the homemade phase-only transparent HIT logo and Merlion logo (see their fabrications in Methods) are shown in Fig. 3a–d, which can effectively reduce the background intensity and capture the high-contrast edge images. By binarization operation, the edge information of the phase image can be easily obtained, as shown in Fig. 3a–d. Meanwhile, Leica reflection DIC module with multiple lenses and prisms is used to image phase-only icons, and the imaging results of large-sized HIT logo and Merlion icon are shown in Supplementary Note 11, which exhibits unidirectional (not isotropic) contrast. However, metasurface-assisted i-DIC microscopy only uses a single-layer structure without the complex combination of lenses and prisms and can capture the phase variance along all directions simultaneously. The results show impressive single-shot, isotropic phase-based optical imaging capability, which significantly outperforms the current DIC microscopy.

We further demonstrate the particle tracking with our i-DIC, which plays key roles in studying biological processes ranging from cellular motion to targeted drug delivery. Conventional methods analyze cellular dynamics by first recording a video and then extracting the translational trajectories using analysis algorithms. The bright-field optical microscopy is usually limited by strong background scattering and interference from out-of-focus light, which is powerless against transparent particles. Our i-DIC microscopy can detect the particles whose refractive index is noticeably distinct from the surroundings. Meanwhile, it can obtain edge information in all directions of the sample without data processing, hence we can directly monitor the moving target in real-time. Taking silicon dioxide (SiO_2_) microspheres (diameter around 20 μm) as an example, we employ our i-DIC microscopy to capture videos of moving SiO_2_ microspheres. The motion tracking of SiO_2_ microspheres is shown in Fig. 3e, by temporal color code using sequences of pictures spaced 0.8 s apart in Supplementary Movie 1. The sequence and direction of motion for each particle can be obtained clearly. The comparative observation using a-DIC and i-DIC microscopy is shown in Supplementary Movie 2. With the edge enhancement, the position, shape, and dynamics of the particles are well revealed, which is the key procedure of label-free particle tracking. We can expect this original technology to be applied in particle-cell interactions, cell dynamics, etc.

### High-resolution i-DIC imaging in biological samples

**Fig. 4:**
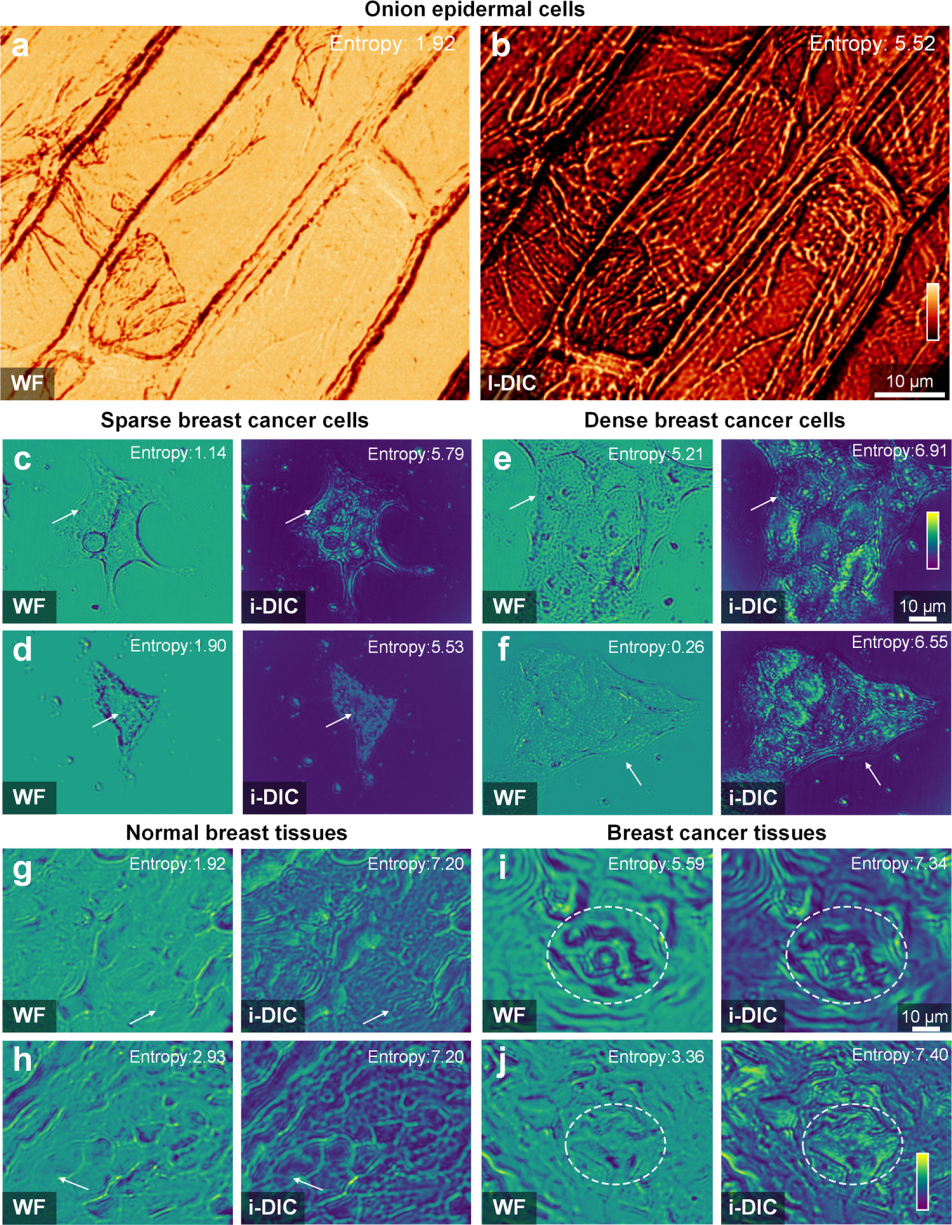
Cells and tissues imaging with i-DIC microscopy. WF widefield. The imaging results of onion epidermal cells in widefield (**a**) and i-DIC (**b**) microscopies. **c**, **d** Sparse breast cancer cells in widefield (left) and i-DIC (right) microscopies. **e**, **f** Dense breast cancer cells in widefield (left) and i-DIC (right) microscopy. **g**, **h** Normal breast tissues in widefield (left) and i-DIC (right) microscopies. **i**, **j** Breast cancer tissues in widefield (left) and i-DIC (right) microscopies.

Apart from inspecting industrial products, DIC microscopy has more crucial applications in life sciences, as it can directly detect live specimens having slight variations in refractive index. To demonstrate the bioimaging performance of the i-DIC microscopy, we apply it in observing onion epidermal cells, and the widefield and i-DIC images are shown in Fig. 4a, b, respectively. The internal structures of cells are less discernible due to the weak absorption properties of the transparent specimen under widefield microscopy, while i-DIC microscopy can effectively reduce the background intensity and capture the high-contrast cellular image.

The imaging of human cells and tissues is pivotal to biological research and medical diagnosis. Clinicians and biologists continue to pursue the visualization of cancer cells and cancer nests more clearly for the screening and treatment of cancers. As cancer cells induce local variations of refractive index, the phase gradient is generated and thus i-DIC microscopy can directly process and detect without staining and labeling. Here, we applied our i-DIC microscopy to observe unstained breast cancer tissues and cells. The sample preparation process is described in Methods. Figure 4c–j show the widefield and i-DIC imaging results of breast cancer cells (Fig. 4c–f) and breast cancer tissues (Fig. 4g–j). Compared with widefield microscopy, our method exhibits clearer and stronger signals at the cell boundaries and intracellular details, indicating exceptionally high sensitivity and precision to detect transparent biological specimens. To be specific, in the observation of sparse breast cancer cells (Fig. 4c, d), the outline of the cell nucleus and cell membrane is clearer as shown by the arrow. For dense breast cancer cells (Fig. 4e, f), the i-DIC microscopy can more clearly delineate the boundaries of cells and segment each cell. In the visualization of breast tissues (Fig. 4g–j), the location and morphology of cancer nests are important references for cancer staging and subsequent treatment. Our recipe can directly help to locate and identify the cancer nests without staining and fluorescence labeling, and the contrast between cancer nests and paracancerous tissue is stronger, as shown in the circle (Fig. 4i, j). We quantify the information capacity contained in widefield images and i-DIC images by calculating the information entropy of the images (definition in Supplementary Note 12)^48^, which reflects how much information is in the picture. The corresponding entropy values are marked at the top-right corners of the pictures in Fig. 4, and the information capacity contained in i-DIC imaging is greater than that in widefield imaging for the same tissue and cell samples.

## Discussion

To summarize, we synergize the multifunctional optical field modulations of a single-layer metasurface with conventional microscopy and developed the single-shot 2*f* i-DIC system. A miniaturized biological imaging and cancer screening system can be constructed for in-vivo diagnosis, which may allow to observe natural cells and tissues in real-time and provides in-situ biomedical observation. Our method can visualize the transparent samples with isotropic contrast and opens an avenue for applications in areas such as biological imaging, early-stage diagnosis, particle tracking, and edge detection.

## Methods

### Tissue and cell use

The study followed the tenets of the Declaration of Helsinki and was approved by the medical ethics committee of the Third Affiliated Hospital of Harbin Medical University.

### Simulations

The transmission spectra are calculated using the frequency domain solver of CST Microwave Studio. The complex refractive index of Si is from Aspnes and Studna 1983: *n*, *k* 0.21–0.83 µm. The refractive index is set to 3.89 and the extinction coefficient is 0.02 at 620 nm. The refractive index of Al_2_O_3_ is from Malitson and Dodge 1972: α-Al_2_O_3_ (Sapphire); *n*(o) 0.20–5.0 µm. The refractive index is set to 1.77. The Si nanofins with the height of 360 nm are modeled as a periodic unit cell (lattice constant 300 nm) on the Al_2_O_3_ substrate.

### Metasurface fabrication

The film of single crystalline silicon with a thickness of 360 nm was first grown on a double-side polished sapphire (Latech Scientific Supply Pte. Ltd.). Then hydrogen silsesquioxane (HSQ, Dow Corning XR-1541-006) was spin-coated onto the substrate at the speed of 4000 revolutions per minute (RPM). The mask of metasurface on HSQ was fabricated by electron beam lithography (eLine Plus, Raith GmbH) at 30 kV acceleration voltage and 360 pA beam current with a 100 × 100 μm^2^ write field. The exposed samples were developed in 25% tetramethylammonium hydroxide (TMAH) solution for 2 min at room temperature (25 °C), followed by rinsing in deionized water for 20 s and dipping in isopropyl alcohol (IPA) solution for 10 s, then blow-dried using nitrogen gas. Afterward, inductively coupled plasma-reactive ion etching (ICP-RIE, Apex SLR ICP, Advance Vacuum Systems) was used to transfer the pattern into silicon film. A carbon tetrafluoride (CF4) dry-etch was first performed at 45 sccm (standard cubic centimeters per minute) gas flow for 5 s with 100 W ICP power and 100 W bias power to remove the surface oxidization layer, then hydrogen bromide (HBr) was applied with a flow rate of 100 sccm, 400 W ICP power, 100 W bias power to etch the silicon at the speed of 83 nm/min. The substrate table temperature was set as 20 °C and the chamber pressure was 10 mTorr during the etching process. Finally, the samples were immersed into hydrofluoric (HF) acid (10%) for 15 s to remove the residue HSQ mask, then cleaned with deionized water and blow-dried with nitrogen gas.

### Fabrication of Icon

The icon samples were fabricated via direct laser writing in the photoresist. The positive photoresist AZ400K was spin-coated onto the fused-silica substrate at the speed of 3000 RPM. Subsequently, the designed patterns were exposed using direct laser writing (Heidelberg Instruments DWL 66+). Finally, the exposed samples were developed in AZ 400K Developer for 2 min at room temperature (25 °C) and blow-dried using nitrogen gas.

### Experimental setup

A red LED light source (GCI-060401, *λ* = 620 ± 10 nm, Daheng Optics Co. Ltd., China) is collimated by a collimating lens (AC127-030-A, Thorlabs, USA) to generate the monochromatic plane wave. The parallel light passes through polarizer 1 (LPVISC100-MP2, Thorlabs, USA) to produce monochromatic polarized light with a polarization direction of 45° to illuminate samples. Metasurface is used to image the sample, and the object distance *a* and image distance *b* should satisfy the Gaussian formula 1*/a* + 1*/b* = 1*/f*. The objective lens (95MM M Plan Apo HL 50× NA 0.42 Donglilai Optics Electronics Enterprise Co., LTD.) and tube lens (Zoom Lens Series 10, Donglilai Optics&Electronics Enterprise Co. LTD, China) form a secondary imaging system to visualize the images produced by metasurface. Polarizer 2 (LPVISC100-MP2, Thorlabs, USA) is placed along the −45° and the DIC image is captured by a CMOS camera (DCU224C, Thorlabs, USA) via Thorcam 3.7.0 software, and the images is processed by ImageJ v1.53 g.

### Preparation of tissue and cell samples

Fresh breast cancer tissues were fixed in 10% paraformaldehyde for at least 24 h. Afterwards, the fixative was removed, and the tissues were transferred into the dehydration box. Then, breast cancer tissues were sequentially immersed in the solutions for dehydration: 70% alcohol for 4 h, 80% alcohol for 2 h, 90% alcohol for 2 h, 95% alcohol for 1 h, anhydrous ethanol for 30 min, another anhydrous ethanol for 30 min, alcohol benzene for 10 min, xylene I for 10 min, xylene II for 10 min, wax I for 1 h, wax II for 1 h, and finally wax III for 1 h to ensure that the moisture in the tissues was completely removed. The wax-soaked tissues were embedded in the embedding machine according to the requirements. The melted wax was put into the embedding box and the breast cancer tissues were taken out from the dehydration box before the wax solidified. Then, we embedded the tissues in paraffin wax and cooled them at 20 °C until they became wax blocks. After the wax solidified, the wax block was removed from the embedding frame and repaired. Next, we sliced the finished wax block on a paraffin slicer with a thickness of 4 μm. The slices were floated in 40 °C warm water of the spreading machine to flatten the tissues. The tissues were picked up with slides and baked in the oven at 60 °C. Waiting for the water to dry and the wax to melt, then take them out and store them at room temperature. T47D breast cancer cells were purchased from the Type Culture Collection of the Chinese Academy of Sciences and cultured in Dulbecco’s modified Eagle’s minimal essential medium with 10% heat-inactivated fetal bovine serum and 1% penicillin/streptomycin at 37 °C in a humid atmosphere with 5% CO_2_. The cells at the logarithmic growth stage were fixed in paraformaldehyde for 30 min, washed with phosphate buffer saline, and dried naturally.

### Statistics and reproducibility

Experiment tissues, cells and imaging region of interests were randomly chosen without prior bias, and data analysis was performed using the same method. All imaging results were repeated with more than 4 times and could be repeated each time. To show the imaging performance, representative imaging results were included in the manuscript.

### Reporting summary

Further information on research design is available in the Nature Portfolio Reporting Summary linked to this article.

## Supplementary information

Supplementary Information

Description of Additional Supplementary Files

Supplementary Movie 1

Supplementary Movie 2

Reporting Summary

## Data Availability

All data generated and analyzed are included in the paper and its supplementary information.

## Code availability

The codes that support the findings of this study are available from the corresponding authors upon request.
