# Supplementary material for: Single-shot isotropic differential interference contrast microscopy
Source: Nat Commun. 2023 Apr 12;14:2063. doi: 10.1038/s41467-023-37606-6 (PMC10097662; doi:10.1038/s41467-023-37606-6)
Supplement: Supplementary file 1 — Supplementary Information [file 41467_2023_37606_MOESM1_ESM.pdf]

Supplementary Information for

Single-shot Isotropic Differential Interference Contrast Microscopy

Content

Supplementary Note 1. Diagram of the conventional DIC microscopy

Supplementary Note 2. Theoretical analysis of the a-DIC microscopy based on single-layer metasurface

Supplementary Note 3. Theoretical analysis of the i-DIC microscopy based on single-layer metasurface

Supplementary Note 4. Simulation results of a-DIC and i-DIC microscopy

Supplementary Note 5. Simulated imaging results of specific samples

Supplementary Note 6. The design of the unit cells for metasurface

Supplementary Note 7. The optical setup for focal spots distribution measurement

Supplementary Note 8. The simulated and measured focal spots distribution

Supplementary Note 9. Theoretical analysis of the edge detection effect for amplitude samples

Supplementary Note 10. The imaging results of the icons, numbers and letters

Supplementary Note 11. The conventional DIC imaging results of the icons

Supplementary Note 12. Image entropy

Supplementary Note 1. Diagram of the conventional DIC microscopy

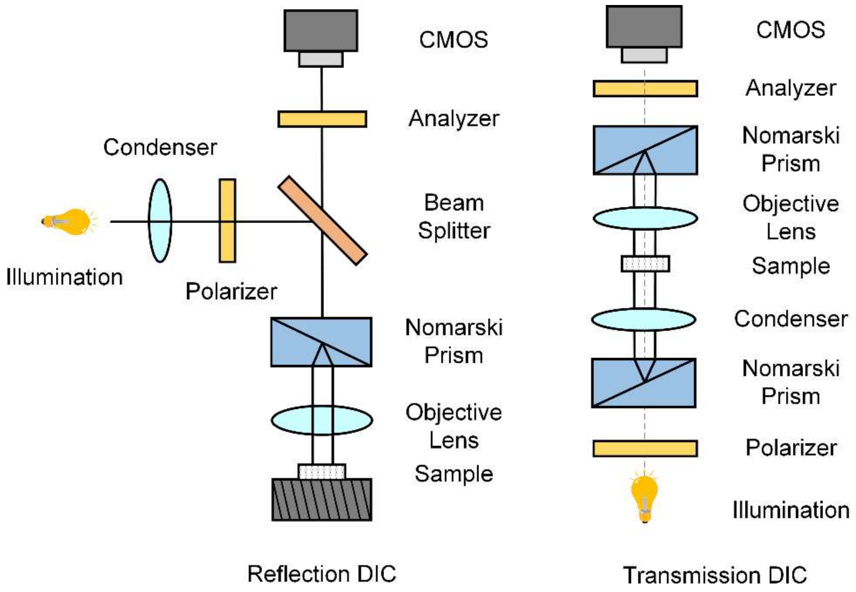

Supplementary Figure 1. The schematic diagram of reflection and transmission DIC microscopy.

In a Wollaston (or Nomarski) prism, incoming light is separated into two linearly polarized beams, which are physically displaced from each other at the output plane (by a distance less than the diffraction limit). Although the two beams propagate through the sample very close to each other, they do not interfere as they have different polarizations. However, each beam takes a slightly different path through the sample, thus a phase difference between the two beams will be endowed. The greater the difference in optical path length between the two closely spaced beams, the greater the contrast will be in the image. After passing through the sample, the two beams are sent through a second prism and analyzer whose polarization direction is perpendicular to the polarizer. Two different polarized waves are projected along the direction of analyzer, which causes two beams to have the same polarization. Therefore, they are able to interfere leading to contrast in the image. As the two beams are physically close to each other through the sample, it is effectively looking at the gradient of the path difference rather than the absolute path difference between the paths. In order to ensure the light for imaging having interacted with the sample, the pair of Wollaston prisms are mounted between two crossed polarizers. Only the light that has experienced a slight polarization change as it passes through the optical system and sample will then be visible.

Supplementary Note 2. Theoretical analysis of the a-DIC microscopy based on single-layer metasurface

In DIC microscopy, the image is formed from the interference between the complex amplitudes of two orthogonal polarized waves that are phase-shifted and carrying lateral shear relative to each other. In this sense, DIC microscopy requires two orthogonally polarized beams that can be modulated independently. A lateral offset (called the shear) and an axial phase-shift (called the bias) need to be formed between these two beams. The bias can be established via appending different phase shifts to the two polarized waves. In the following part, we analyze how to construct the phase distribution to introduce the transverse shear.

Assuming that the pupil plane coordinates are (ξ, η), and the introduced shear is $2\Delta s$, while the introduced bias retardation is $2\Delta\varphi$. As shown in the supplementary Figure 2, according to the generalized Snell's laws[1]: $\sin(\theta_t)n_t - \sin(\theta_i)n_i = \frac{\lambda}{2\pi}\frac{d\Phi}{d\xi}$, in order to separate two orthogonally polarized beams, the add-on abrupt phase shift should be $\pm\frac{2\pi\Delta s}{\lambda f}\xi$, where positive and negative signs correspond to *x*- and *y*-polarizations, respectively. Therein f is the focal length of the metasurface, and λ is the wavelength

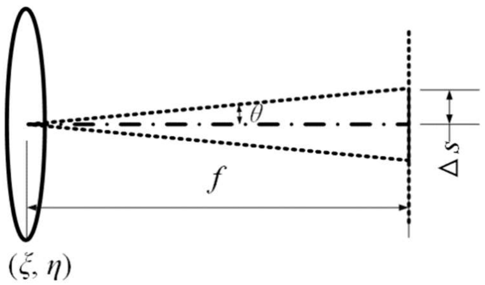

Supplementary Figure 2. The schematic diagram of lateral separation of two orthogonally polarized beams.

Similar to the conventional Normarski DIC microscopy, we can exploit the multifunctional optical field modulations of metasurface to implement a-DIC microscopy. The polarization conversion and information change of the optical field

are illustrated in the polarization coordinate system, as seen in Supplementary Figure 3**a**. Assuming that the object's complex amplitude transmission function is $O(x, y)$ in the cartesian coordinate system, coherent illumination, ideal imaging system, perfect polarized components, and there is no instrumental stray light, and the polarizer and analyzer align to ±45°, respectively. The optical path schematic of metasurface-based a-DIC microscopy is shown in Supplementary Figure 3**b**. Using the plane wave to illuminate the object, the transmitted light $O(x, y)$ passes through the polarizer and becomes a linearly polarized beam at 45°, which can be decomposed into equi-amplitude linear superpositions of x- and y-polarizations. The metasurface can produce a laterally displaced copy with a bias phase of the sample for x- and y-polarizations, respectively. More specifically, the ideal imaging results are $O(x\pm\Delta s, y)\exp(\pm i\Delta\varphi)$, where positive and negative signs correspond to x- and y-polarizations, respectively. In view of the analyzer, the image results generated by the metasurface are projected at the -45°, and the image sensor captures polarization interference between x- and y- polarization waves, which can be described as

$$I_{A-DIC} \propto \left| O(x-\Delta s, y)e^{-i\Delta\varphi} \text{-} O(x+\Delta s, y)e^{i\Delta\varphi} \right|^2 \tag{1}$$

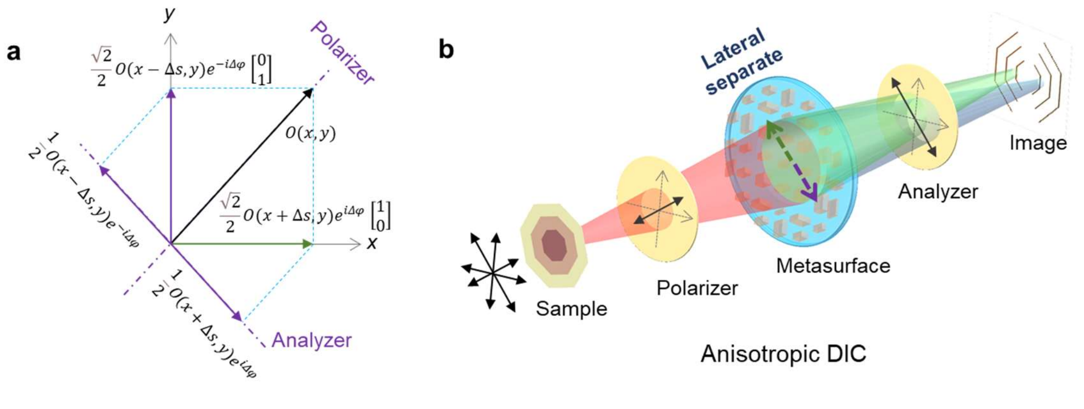

Supplementary Figure 3. Schematic of metasurface-based a-DIC microscopy and its operation principle. **a**. The principle schematic diagram of a-DIC microscopy. **b**. The optical path schematic of metasurface-based a-DIC microscopy.

In order to realize the a-DIC microscopy using a flat metasurface, similar to the conventional DIC microscopy, the elaborated metasurface should possess three abilities: (1) optical imaging features, (2) polarization beam transverse separation

(shear), (3) polarization beam axial phase-shift (bias). Therein the optical imaging characteristics can be satisfied by attaching to the quadratic-phase distribution. Hence, considering the symmetry, for *x*- and *y*-polarization, the introduced abrupt phase shifts can be written as

$$\phi_x(\xi,\eta) = -\frac{\pi}{\lambda f}(\xi^2+\eta^2) + \frac{2\pi\Delta s}{\lambda f}\xi - \Delta\varphi \tag{2}$$

$$\phi_y(\xi,\eta) = -\frac{\pi}{\lambda f}(\xi^2+\eta^2) - \frac{2\pi\Delta s}{\lambda f}\xi + \Delta\varphi \tag{3}$$

where $-\frac{\pi}{\lambda f}(\xi^2+\eta^2)$ is the imaging phase, $\pm\frac{2\pi\Delta s}{\lambda f}\xi$ is the shear phase, and $\pm\Delta\varphi$ is the bias phase.

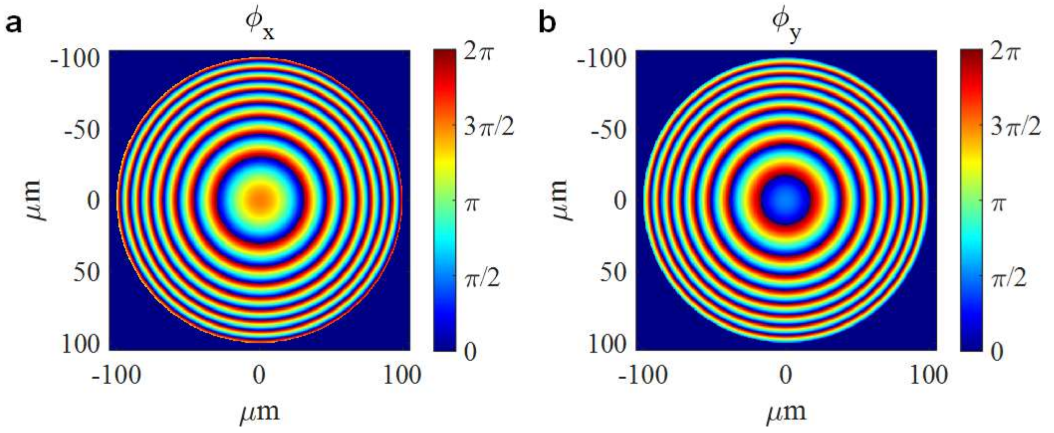

Supplementary Figure 4. The required phase distributions of a-DIC metasurface in **a** *x*-polarization and **b** *y*-polarization. The pupil radius of metasurface is 100 μm. The wavelength λ = 620 nm. The focus f = 1 mm. The shear 2Δs = 1 μm. The bias 2Δφ = π.

Next, considering the non-ideal imaging case with the limitation of optical pupil size, the transmission functions of the proposed metasurface for *x*- and *y*-polarization, respectively can be written as

$$\begin{aligned} t_x(\xi,\eta) &= P(\xi,\eta)\exp\left[i\phi_x(\xi,\eta)\right] \\ &= P(\xi,\eta)\exp\left\{-i\left[\frac{\pi}{\lambda f}(\xi^2+\eta^2) - \frac{2\pi\Delta s}{\lambda f}\xi + \Delta\varphi\right]\right\} \end{aligned} \tag{4}$$

$$
\begin{aligned}
t_y(\xi,\eta) &= P(\xi,\eta)\exp\left[i\phi_y(\xi,\eta)\right] \\
&= P(\xi,\eta)\exp\left\{-i\left[\frac{\pi}{\lambda f}(\xi^2+\eta^2)+\frac{2\pi\Delta s}{\lambda f}\xi-\Delta\varphi\right]\right\}
\end{aligned} \tag{5}
$$

where $P(\xi,\eta)=\begin{cases}1, & \sqrt{\xi^2+\eta^2}\le a \\ 0, & others\end{cases}$ (a is pupil radius) represents the finite optical aperture limitation of the metasurface. The calculation can be found in Supplementary Figure 4.

Without regard to a pair of crossed polarizers, for x-polarization, the generalized pupil function or coherent transfer function (CTF)[2] of the metasurface-based a-DIC system can be regarded as $P(\xi,\eta)\exp\left[i\left(\frac{2\pi\Delta s}{\lambda f}\xi-\Delta\varphi\right)\right]$.

The amplitude point spread function (APSF)[3] can be calculated by Fourier transform of the CTF

$$
\begin{aligned}
h_x(x,y) &= \iint_{-\infty}^{\infty} P(\xi,\eta)\exp\left[i\left(\frac{2\pi\Delta s}{\lambda f}\xi-\Delta\varphi\right)\right]\exp\left[-i\frac{2\pi}{\lambda f}(x\xi+y\eta)\right]d\xi d\eta \\
&= e^{-i\Delta\varphi}k(x-\Delta s,y)
\end{aligned} \tag{6}
$$

where $k(x, y)$ is the APSF of widefield microscopy under coherent illumination, which can be written as Fourier transform of pupil function

$$
k(x,y)=\iint_{-\infty}^{\infty} P(\xi,\eta)\exp\left[-i\frac{2\pi}{\lambda f}(x\xi+y\eta)\right]d\xi d\eta \tag{7}
$$

Similarly, for y-polarization, without regard to a pair of crossed polarizers, the generalized pupil function or CTF of the system can be regarded as $P(\xi,\eta)\exp\left[-i\left(\frac{2\pi\Delta s}{\lambda f}\xi-\Delta\varphi\right)\right]$. The APSF under the condition of y-polarization can be written as

$$
h_y(x,y)=e^{i\Delta\varphi}k(x+\Delta s,y) \tag{8}
$$

Since the polarization direction of the second polarizer is along -45°, the imaging results with two orthogonally polarizations generated by the metasurface are projected along this direction. The output complex amplitude of optical field can be shown as

$$\begin{aligned} A(x,y) &= O(x,y)\otimes h_x(x,y) - O(x,y)\otimes h_y(x,y) \\ &= O(x,y)\otimes\left[e^{-i\Delta\varphi}k(x-\Delta s,y)\text{-}e^{i\Delta\varphi}k(x+\Delta s,y)\right] \end{aligned} \tag{9}$$

The comprehensive APSF of the metasurface-based a-DIC system can be expressed as

$$h(x,y) = e^{-i\Delta\varphi}k(x-\Delta s,y)\text{-}e^{i\Delta\varphi}k(x+\Delta s,y) \tag{10}$$

For the coherent optical imaging system, the output intensity of the optical field can be written as

$$\begin{aligned} I(x,y) &= \left|O(x,y)\otimes h(x,y)\right|^2 \\ &= \left|O(x,y)\otimes\left[e^{-i\Delta\varphi}k(x-\Delta s,y)\text{-}e^{i\Delta\varphi}k(x+\Delta s,y)\right]\right|^2 \end{aligned} \tag{11}$$

When the amplitude contrast is small, we should have $|h(x,y)| = 2|k(x,y)\sin(\Delta\varphi)|$. If the incident image is purely amplitude and $\Delta\varphi$ is induced by the metasurface, we thus require that $2\Delta\varphi = 2l\pi$, $l = 0, \pm1, ...$ In this way, two images are overlapped, till have destructive interference. Nevertheless, if $\Delta\varphi$ is purely induced by the phase difference from the sample, we should have $\Delta\varphi = \nabla_x\varphi\cdot\frac{\Delta s}{2}$, which hence is the "optical differentiation". In this case, $2\Delta\varphi = \pi(2l+1)$, $l = 0, \pm1$ ... should give the best contrast, because at the output point (x, y), we have the strongest signals in the position where the refractive index changes.

The CTF of the metasurface-based a-DIC system can be calculated by inverse Fourier transform of the APSF

$$H(\xi,\eta) = \mathcal{F}^{-1}\{h(x,y)\} = -j\sin(2\pi\xi\Delta s + \Delta\varphi)P(\xi,\eta) \tag{12}$$

which is consistent with the CTF of conventional DIC microscopy[4].

Supplementary Note 3. Theoretical analysis of the i-DIC microscopy based on single-layer metasurface

Different from the lateral shear in a-DIC microscopy, the i-DIC microscopy requires the radial shear. According to the generalized Snell's laws[1], analogous to lateral shear, in order to separate two orthogonally polarized beams along the radial direction, the add-on phase gradient should be $\pm\frac{2\pi\Delta s}{\lambda f}\sqrt{\xi^2+\eta^2}$, where positive and negative signs correspond to x- and y-polarizations, respectively.

Similar to the a-DIC microscopy, we can exploit the multifunctional optical field modulations of metasurface to implement i-DIC microscopy. The polarization conversion and information change of the optical field are illustrated in the polarization coordinate system, as seen in Supplementary Figure 5**a**. To analyze the imaging characteristic of the i-DIC microscopy, we convert the cartesian coordinate system into the polar coordinate system. Assuming that the object's complex amplitude transmission function in the polar coordinate system can be written as $O(r, \theta)$, coherent illumination, ideal imaging system, perfect polarized components, and that there is no instrumental stray light, and the polarizer and analyzer align to ±45°, respectively. The optical path schematic of metasurface-based i-DIC microscopy is shown in Supplementary Figure 5**b**. Using the plane wave to illuminate the object, the transmitted light $O(r, \theta)$ passes through the polarizer and becomes a linearly polarized beam at 45°, which can be decomposed into equi-amplitude linear superpositions of x- and y-polarizations. The metasurface can produce a radial displaced copy with a bias phase of the sample for x- and y-polarizations, respectively. More specifically, the ideal imaging results are $O(r\pm\Delta s, \theta)\exp(\pm i\Delta\varphi)$, where positive and negative signs correspond to x- and y-polarizations, respectively. In view of the analyzer, the image results generated by the metasurface are projected at the -45°, and the image sensor captures polarization interference between x- and y- polarization waves, which can be described as

$$I_{I-DIC} \propto \left|O(r-\Delta s,\theta)e^{-i\Delta\varphi} - O(r+\Delta s,\theta)e^{i\Delta\varphi}\right|^2 \tag{13}$$

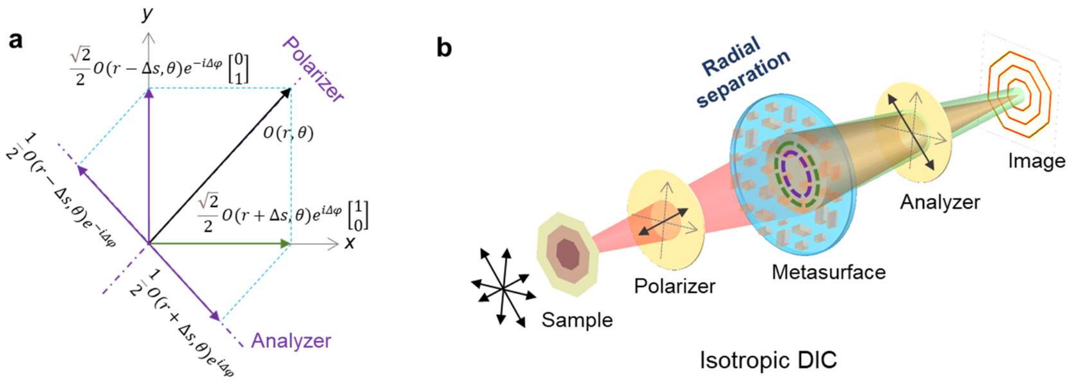

Supplementary Figure 5. Schematic of metasurface-based i-DIC microscopy and its operation principle. **a**. The principle schematic diagram of i-DIC microscopy. **b**. The optical path schematic of metasurface-based i-DIC microscopy.

Similar to a-DIC microscopy and considering the rotational symmetry, for *x*- and *y*-polarizations, the introduced abrupt phase shifts of the metasurface in i-DIC microscopy can be written as

$$\phi_x(\xi,\eta) = -\frac{\pi}{\lambda f}(\xi^2+\eta^2) + \frac{2\pi\Delta s}{\lambda f}\sqrt{\xi^2+\eta^2} - \Delta\varphi \tag{14}$$

$$\phi_y(\xi,\eta) = -\frac{\pi}{\lambda f}(\xi^2+\eta^2) - \frac{2\pi\Delta s}{\lambda f}\sqrt{\xi^2+\eta^2} + \Delta\varphi \tag{15}$$

where $\pm\frac{2\pi\Delta s}{\lambda f}\sqrt{\xi^2+\eta^2}$ is the shear phase, which can split two orthogonally polarized beams along the radial direction. The calculation can be found in Supplementary Figure 6.

The transmission functions of the metasurface for *x*- and *y*-polarization can be expressed as

$$\begin{aligned} t_x(\xi,\eta) &= P(\xi,\eta)\exp\left[i\phi_x(\xi,\eta)\right] \\ &= P(\xi,\eta)\exp\left\{-i\left[\frac{\pi}{\lambda f}(\xi^2+\eta^2) - \frac{2\pi\Delta s}{\lambda f}\sqrt{\xi^2+\eta^2} + \Delta\varphi\right]\right\} \end{aligned} \tag{16}$$

$$\begin{aligned} t_y(\xi,\eta) &= P(\xi,\eta)\exp\left[i\phi_y(\xi,\eta)\right] \\ &= P(\xi,\eta)\exp\left\{-i\left[\frac{\pi}{\lambda f}(\xi^2+\eta^2) + \frac{2\pi\Delta s}{\lambda f}\sqrt{\xi^2+\eta^2} - \Delta\varphi\right]\right\} \end{aligned} \tag{17}$$

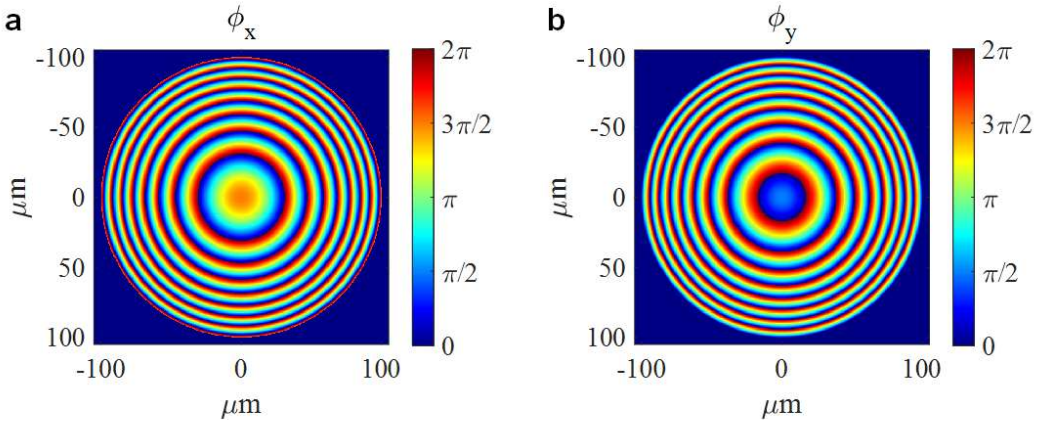

Supplementary Figure 6. The required phase distributions of i-DIC metasurface in **a** *x*-polarization and **b** *y*-polarization. The pupil radius of metasurface is 100 μm. The wavelength λ = 620 nm. The focus f = 1 mm. The shear 2Δs = 1 μm. The bias 2Δφ = π.

Without regard to a pair of crossed polarizers, for *x*-polarization, the generalized pupil function or CTF of the system can be regarded as $P(\xi,\eta)\exp\left[i\left(\frac{2\pi\Delta s}{\lambda f}\sqrt{\xi^2+\eta^2}-\Delta\varphi\right)\right]$. Correspondingly, the APSF can be calculated as

$$h_x(x,y)=\iint_{-\infty}^{\infty}P(\xi,\eta)\exp\left[i\left(\frac{2\pi\Delta s}{\lambda f}\sqrt{\xi^2+\eta^2}-\Delta\varphi\right)\right]\exp\left[-i\frac{2\pi}{\lambda f}(x\xi+y\eta)\right]d\xi d\eta \tag{18}$$

By converting the cartesian coordinate system to the polar coordinate system, $\rho=\sqrt{\xi^2+\eta^2}$, $\phi=\arctan(\frac{\xi}{\eta})$, the CTF can be expressed as $P(\rho,\phi)\exp\left[i\left(\frac{2\pi\Delta s}{\lambda f}\rho-\Delta\varphi\right)\right]$ in the polar coordinate system.

In the polar coordinate system, the APSF can be calculated by Fourier-Bessel transform of the CTF

$$\begin{aligned}h_x(r,\theta)&=\int_0^{\infty}\int_0^{2\pi}P(\rho,\phi)\exp\left[i\left(\frac{2\pi\Delta s}{\lambda f}\rho-\Delta\varphi\right)\right]\exp\left[-i\frac{2\pi}{\lambda f}\rho r(\cos\theta\cos\phi+\sin\theta\sin\phi)\right]\rho d\rho d\theta\\&=2\pi e^{-i\Delta\varphi}\int_0^{\infty}P(\rho)J_0\left(\frac{2\pi}{\lambda f}\rho(r-\Delta s)\right)\rho d\rho=e^{-i\Delta\varphi}k(r-\Delta s)\end{aligned} \tag{19}$$

where $k(r)$ is the APSF of widefield microscopy in the polar coordinate system, which

can be written as Fourier-Bessel transform of pupil function

$$k(r)=2\pi\int_0^\infty P(\rho)J_0(\frac{2\pi}{\lambda f}r\rho)\rho d\rho \tag{20}$$

Similarly, for y-polarization, the generalized pupil function or CTF of the system can be regarded as $P(\xi,\eta)\exp\left[-i\left(\frac{2\pi\Delta s}{\lambda f}\sqrt{\xi^2+\eta^2}-\Delta\varphi\right)\right]$.

The corresponding APSF under the condition of y-polarization in the polar coordinate system can be expressed as

$$h_y(r,\theta)=e^{i\Delta\varphi}k(r+\Delta s) \tag{21}$$

Considering the analyzer is along the -45° polarization direction, the imaging results with two orthogonally polarizations generated by the metasurface are projected along this direction, hence the final complex amplitude of the optical field can be expressed as

$$\begin{aligned}A(r,\theta)&=O(r,\theta)\otimes h_x(r,\theta)-O(r,\theta)\otimes h_y(r,\theta)\\&=O(r,\theta)\otimes\left[e^{-i\Delta\varphi}k(r-\Delta s)-e^{i\Delta\varphi}k(r+\Delta s)\right]\end{aligned} \tag{22}$$

The comprehensive APSF of the metasurface-based i-DIC system in the polar coordinate system can be expressed

$$h(r)=e^{-i\Delta\varphi}k(r-\Delta s)\text{-}e^{i\Delta\varphi}k(r+\Delta s) \tag{23}$$

For the coherent optical imaging system, the output intensity of the optical field can be written as

$$\begin{aligned}I(r,\theta)&=\left|O(r,\theta)\otimes h(r,\theta)\right|^2\\&=\left|O(r,\theta)\otimes\left[e^{-i\Delta\varphi}k(r-\Delta s,\theta)\text{-}e^{i\Delta\varphi}k(r+\Delta s,\theta)\right]\right|^2\end{aligned} \tag{24}$$

The corresponding CTF of the metasurface-based i-DIC system in polar coordinates can be calculated by inverse Fourier-Bessel transform of the APSF

$$H(\rho)=\mathcal{F}^{-1}\left\{h(r)\right\}=-j\sin(2\pi\rho\Delta s+\Delta\varphi)K(\rho) \tag{25}$$

Different from the classical a-DIC microscopy based on Nomarski or Wollaston prism, whose shear is along the rectilinear direction and generates two polarized images to separate transversely, the shear of our metasurface-based i-DIC microscopy

is along the radial direction, which produces two polarized images of one larger slightly and one smaller slightly. The output results can be described with polarization interference.

Supplementary Note 4. Simulation results of a-DIC and i-DIC microscopy

The bias is the other parameter introduced by metasurface. Next, we analyze the imaging results under different bias conditions via numerical simulation. We take a phase-only target with unity amplitude (undetectable under the conventional wide-field microscopy), which has image information of $O(x,y)=e^{i\phi(x,y)}$. The phase distribution is shown in Supplementary Figure 7a, and the simulated intensity variance is shown in Supplementary Figure 7b-p. The phase variance along y-direction can be visualized while not along the x-direction when using the a-DIC microscopy. On the contrary, the edge information of every direction is preserved in our i-DIC microscopy. Meanwhile, under some conditions (such as Supplementary Figure 7g, h, i, k, and l), the phase variance along y-direction in partial areas is not clear or even visible in a-DIC microscopy, while it has clear contrast under different bias in i-DIC microscopy. Thus, we can choose bias at liberty in i-DIC microscopy.

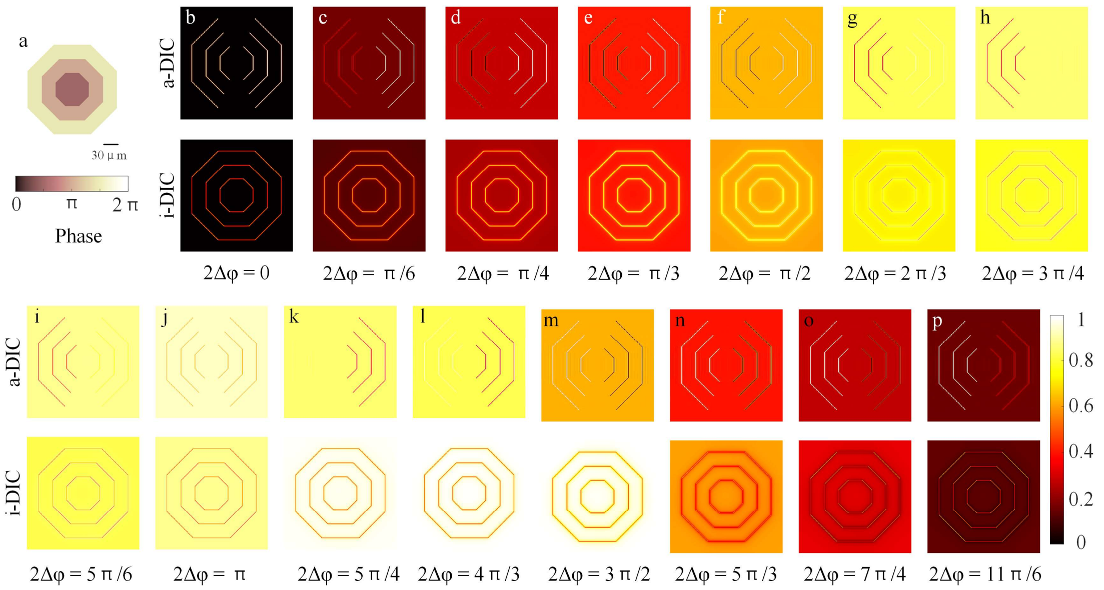

Supplementary Figure 7. The influence of bias in a-DIC and i-DIC microscopy. **a**, The phase distribution of the simulated phase-only target with unity amplitude. **b-p**, The simulated imaging results of different biases in a-DIC (top) and i-DIC (bottom) microscopy. The shear is 0.4 μm, NA = 0.7.

Supplementary Note 5. Simulated imaging results of specific samples

According to the characteristic of DIC microscopy, the point spread functions (PSF) of a-DIC and i-DIC can be shown respectively:

$$h_{a-DIC} = e^{i\Delta\varphi} k(x+\Delta s, y) - e^{-i\Delta\varphi} k(x-\Delta s, y) \tag{26}$$

$$h_{i-DIC} = e^{i\Delta\varphi} k(r+\Delta s) - e^{-i\Delta\varphi} k(r-\Delta s) \tag{27}$$

In the incoherent case, the image intensity is given by the convolution equation

$$I = \left| h * O \right|^2 \tag{28}$$

Without considering the limit of optical diffraction, the PSF can be written as

$$h_{a-DIC} = e^{i\Delta\varphi} \delta(x+\Delta s, y) - e^{-i\Delta\varphi} \delta(x-\Delta s, y) \tag{29}$$

$$h_{i-DIC} = e^{i\Delta\varphi} \delta(r+\Delta s) - e^{-i\Delta\varphi} \delta(r-\Delta s) \tag{30}$$

The image intensity can be described as

$$I_{a-DIC} = \left| h_{a-DIC} * O \right|^2 = \left| e^{i\Delta\varphi} O(x+\Delta s, y) - e^{-i\Delta\varphi} O(x-\Delta s, y) \right|^2 \tag{31}$$

$$I_{i-DIC} = \left| h_{i-DIC} * O \right|^2 = \left| e^{i\Delta\varphi} O(r+\Delta s) - e^{-i\Delta\varphi} O(r-\Delta s) \right|^2 \tag{32}$$

For pure phase object, the transmission function can be written $O(x, y) = e^{i\Phi(x,y)}$ (or $O(r,\theta) = e^{i\Phi(r,\theta)}$ in polar coordinate system). According to first-order Taylor's expansion equation, object function can be approximated as $O(x, y) \approx 1 + \Phi(x, y)$ (or $O(r,\theta) = 1 + \Phi(r,\theta)$ in polar coordinate system). If the bias $2\Delta\varphi = 0$, the intensity of the classical or anisotropic DIC microscopy can be described as

$$\begin{aligned} I_{a-DIC} &\approx \left| \Phi(x+\Delta s, y) - \Phi(x-\Delta s, y) \right|^2 \\ &= 4\Delta s^2 \left| \frac{\Phi(x+\Delta s, y) - \Phi(x-\Delta s, y)}{2\Delta s} \right|^2 \\ &\approx 4\Delta s^2 \left| \frac{\partial \Phi}{\partial x} \right|^2 \end{aligned} \tag{33}$$

In the same way, the intensity of isotropic DIC microscopy can be written as

$$I_{iso} = 4\Delta s^2 \left| \frac{\partial \Phi}{\partial r} \right|^2 \tag{34}$$

Therein, $r = \sqrt{x^2 + y^2}$. On the basis of the derivative rule of compound function,

$$\frac{\partial \Phi}{\partial x} = \frac{\partial \Phi}{\partial r}\frac{\partial r}{\partial x} + \frac{\partial \Phi}{\partial \theta}\frac{\partial \theta}{\partial x} \tag{35}$$

If $\frac{\partial \Phi}{\partial \theta} = 0$

$$\frac{\partial \Phi}{\partial x} = \frac{\partial \Phi}{\partial r}\frac{\partial r}{\partial x} = \frac{\partial \Phi}{\partial r}\frac{x}{\sqrt{x^2 + y^2}} = \frac{\partial \Phi}{\partial r}\cos\theta \le \frac{\partial \Phi}{\partial r} \tag{36}$$

Therefore, we can find that

$$I_{aniso} \le I_{iso} \tag{37}$$

When $\theta = 0$, π, then the equality holds. In this case, i-DIC microscopy has higher intensity than a-DIC microscopy at the same point in the two-dimensional plane except for the points on the x axis. However, in other case, for example $\frac{\partial \Phi}{\partial \theta} \ne 0$, the i-DIC is not necessarily better than a-DIC if only consider the x-direction.

Compared with a-DIC microscopy (Ref. 10-15), i-DIC microscopy can capture the phase variance along all directions simultaneously. It incorporates the advantages of a-DIC microscopy and is more powerful, so compared with a-DIC, it is more suitable for All types of samples including bio-objects.

We can also use some special samples as simulated simulation example. For particularly directional samples, we take diffraction gratings as an example. A phase-type diffractive grating is an optical component with a periodic structure that diffracts light into several beams traveling in different directions (i.e., different diffraction angles), which are commonly used in monochromators and spectrometers, but other applications are also possible such as optical encoders for high precision motion control and wavefront measurement. Therefore, the imaging and measurement of the diffraction grating are of great significance. The shear is along x direction in a-DIC microscopy, and the imaging results of diffraction grating using a-DIC and i-DIC microscopy are shown in Supplementary Figures 8. If the shear direction is parallel to the grating direction, the image intensities in a-DIC and i-DIC microscopy are the

same. However, If the shear direction is perpendicular to the grating direction, the imaging result of a-DIC microscopy is blank, and there is no effective information. On the contrary, the shape and boundary are clearly visible in i-DIC microscopy.

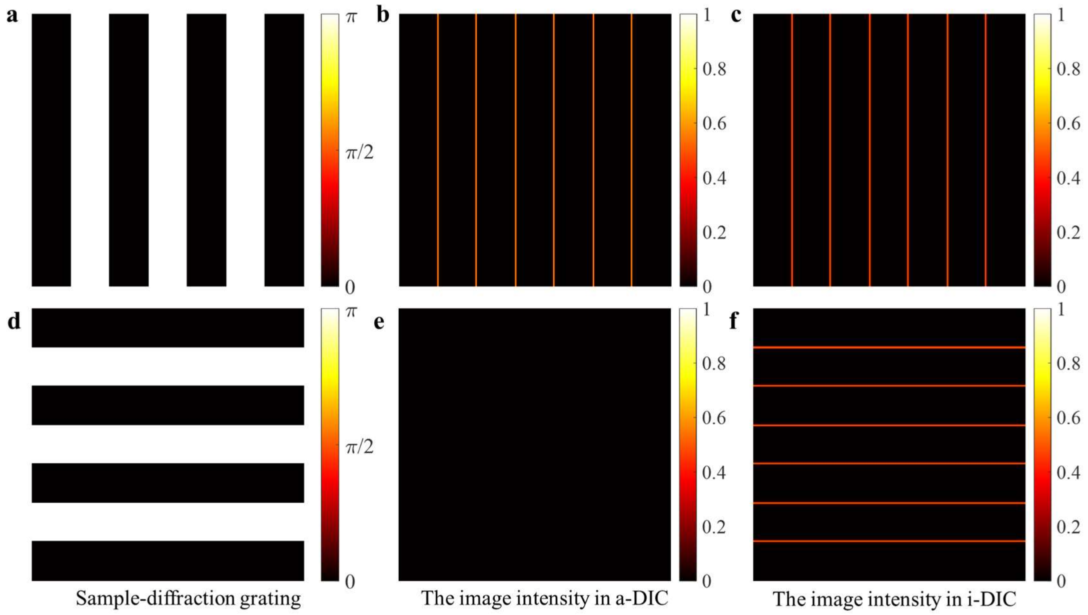

Supplementary Figure 8. The diffraction gratings and normalization intensity of a-DIC and i-DIC microscopy. **a**. The phase map of the diffraction gratings, the grating direction is along *x* direction. **b**. The image intensity of diffraction gratings using a-DIC microscopy. the shear is along *x* direction. **c**. The image intensity of diffraction gratings using i-DIC microscopy. **d**. The phase map of the diffraction gratings, the grating direction is along *y* direction. **e**. The image intensity of diffraction gratings using a-DIC microscopy. the shear is along *x* direction. **f**. The image intensity of diffraction gratings using i-DIC microscopy.

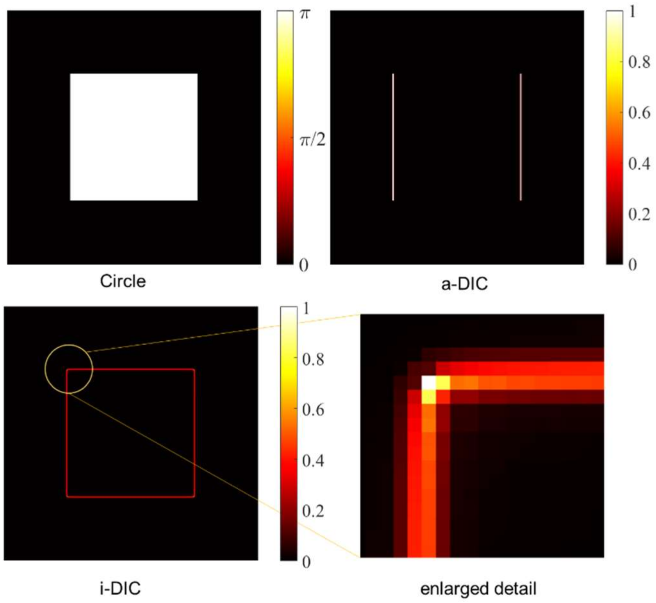

Supplementary Figure 9. The square sample and normalization intensity of a-DIC and i-DIC microscopy.

Meanwhile, for rectangular or square objects with sharp right-angle corners, we take the square sample as an example. The shear is along x direction in a-DIC microscopy, so the edge contrast of the a-DIC image is strongest in the x direction while disappears in the y direction. This will cause the loss of some orientation information, which is not conducive to image restoration. On the contrary, the edge contrast of the i-DIC image remains constant in all directions. More **importantly, sharp corner points in i-DIC imaging are brighter than the surrounding edges**, as shown in enlarged detail of Figure R5, which can be used for corner recognition and detection. Thus, compared with classical a-DIC microscopy, i-DIC microscopy not only retain the edge information of other directions, but also can obtain additional performance, e.g., corner detection.

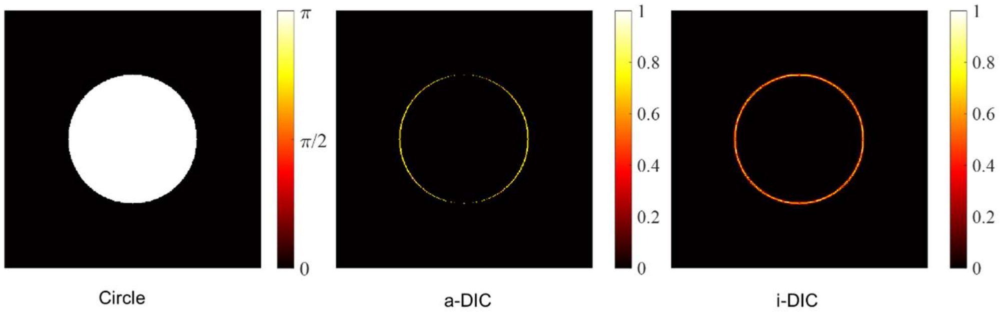

Supplementary Figure 10. The circle and normalization intensity with a-DIC and i-DIC microscopy.

For samples that are not directional, we take a circle as an example. The imaging results of circle using a-DIC and i-DIC microscopy are shown in Supplementary Figure 10. The shear is along x direction in a-DIC microscopy, so the image intensity is not uniform in each direction. The edge contrast of the a-DIC image is strongest in the x direction, decreases in other directions and disappears in the y direction. This will cause the loss of some orientation information, which is not conducive to image restoration. On the contrary, the edge contrast of the i-DIC image remains constant in all directions.

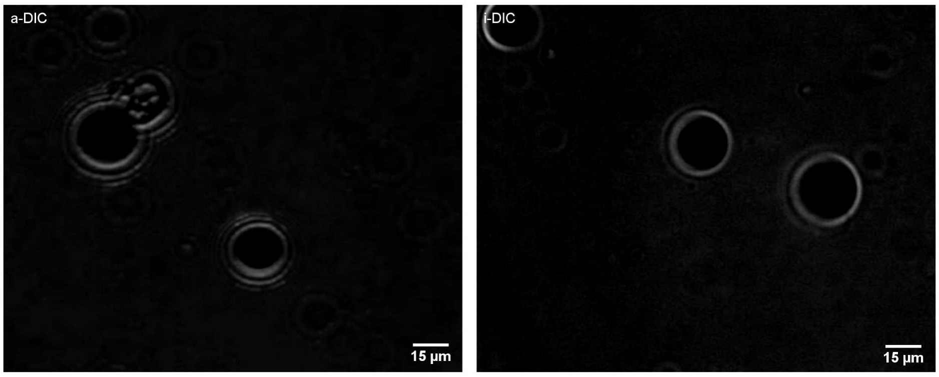

Supplementary Figure 11. The moving SiO_2 microspheres imaging results of a-DIC and i-DIC microscopy.

We employ our metasurface-enabled DIC metasurface to observe silicon dioxide (SiO_2) microspheres in motion, as shown in **Supplementary Movie 2**. A screenshot from the video is shown in Supplementary Figure 11. As seen that the edge in all directions of SiO_2 microspheres was visible clearly in dark background in i-DIC microscopy while the edge contrast of the a-DIC microscopy is strongest in the y direction, which is consistent with the simulation results (Supplementary Figure 10).

In a word, for samples that are or are not directional, our i-DIC microscopy can also perform better. Compared with a-DIC microscopy, i-DIC would be more general with those simulations (and experimental comparisons) provided.

Supplementary Note 6. The design of the unit cells for metasurface

To achieve the desired independent phase control of two orthogonally polarized waves, we use anisotropic monocrystalline silicon (Si) nanofins on a sapphire (Al_2O_3) substrate as the unit cell shown in Supplementary Figure 12, for which the height of nanofins is 360 nm, and the period of the structure is 300 nm, the width and length of the nanofins can be adjusted to satisfy different phase profiles. CST Microwave Studio is used to simulate the transmission coefficient under *x*- and *y*-polarization for unit cells with different parameters. The simulated transmission coefficients are shown in Supplementary Figure. 13, operating at 620 nm.

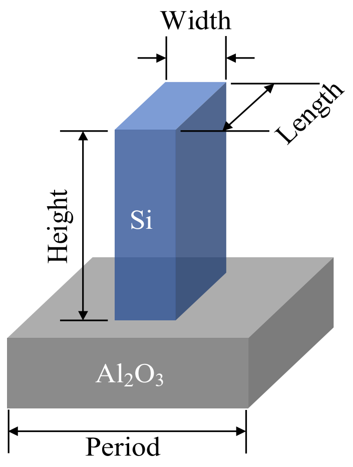

Supplementary Figure 12. The unit cell structure for the metasurface.

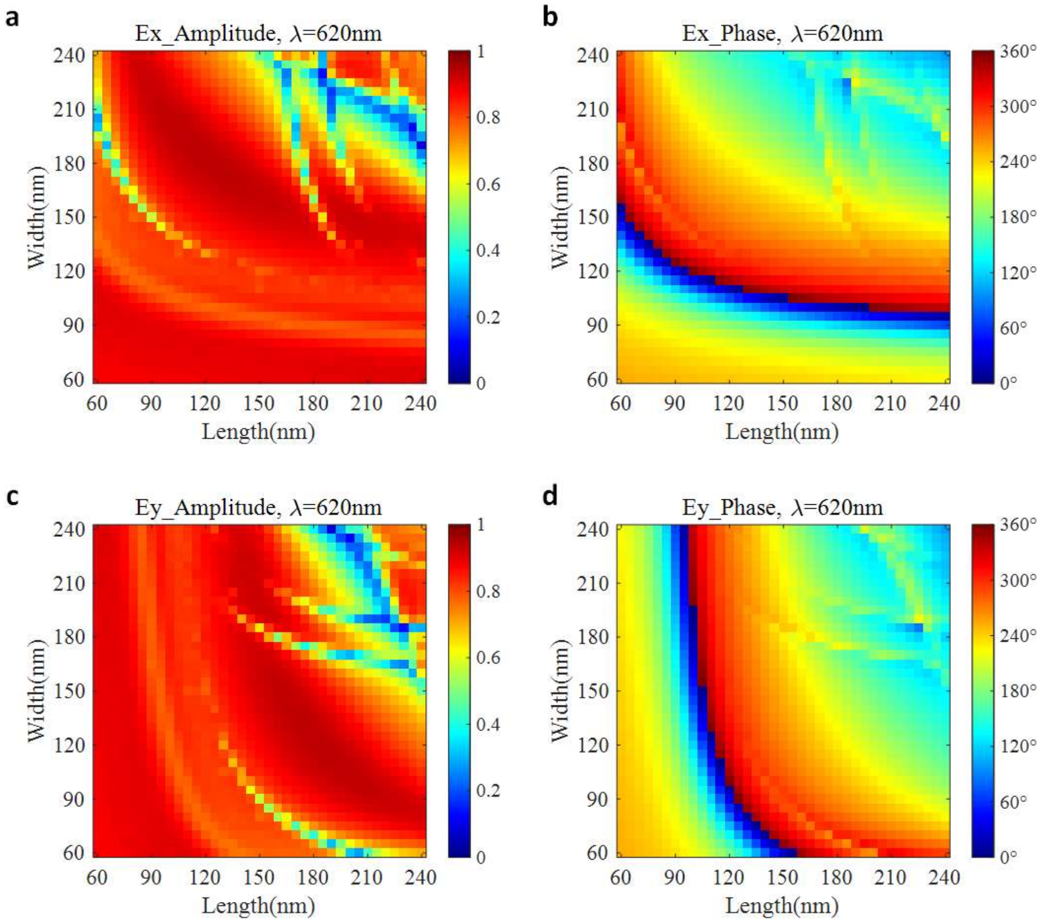

Supplementary Figure 13. The simulated transmission amplitude and transmission phase of the *x*- and *y*-polarizations for silicon nanofins with different widths and lengths, operating at λ= 620 nm., the simulated transmission **a** amplitude and **b** phase for *x*-polarization. The simulated transmission **c** amplitude and **d** phase for *y*-polarization.

Supplementary Note 7. The optical setup for focal spots distribution measurement

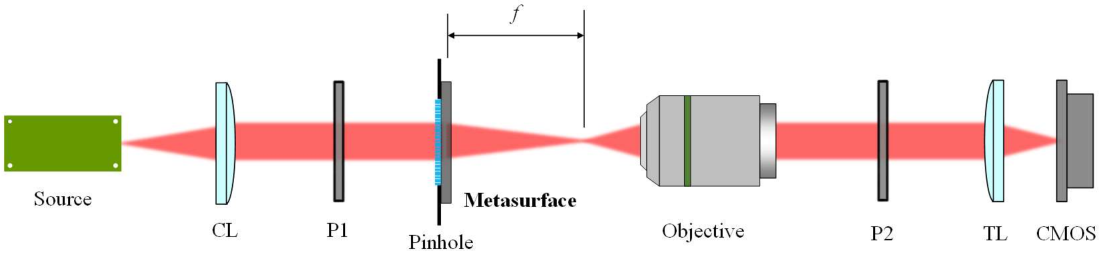

Supplementary Figure 14. The optical setup for focal spots distribution measurement. CL, collimating lens. P1, P2, a pair of crossed polarizers. TL, tube lens. CMOS, complementary metal oxide semiconductor. A red LED light source (GCI-060401, Daheng Optics Co. Ltd., China) is collimated by a collimating lens (AC127-030-A, Thorlabs, USA) to generate a monochromatic plane wave. The parallel light passes through polarizer 1 (LPVISC100-MP2, Thorlabs, USA) to produce a monochromatic polarized wave with a polarization direction of 45° to illuminate the metasurface. The phase profiles for two orthogonal polarizations can be found in Supplementary Note 1 and Supplementary Note 2. The objective lens (95MM M Plan Apo HL 50X, Donglilai Optics & Electronics Enterprise Co. LTD, China) and tube lens (Zoom Lens Series 10, Donglilai Optics & Electronics Enterprise Co. LTD, China) form a secondary imaging system to visualize the focal spot produced by the metasurface. Polarizer 2 (LPVISC100-MP2, Thorlabs, USA) is placed along the -45° and the focal spot is captured by a CMOS camera (DCU224C, Thorlabs, USA).

Supplementary Note 8. The simulated and measured focal spots distribution

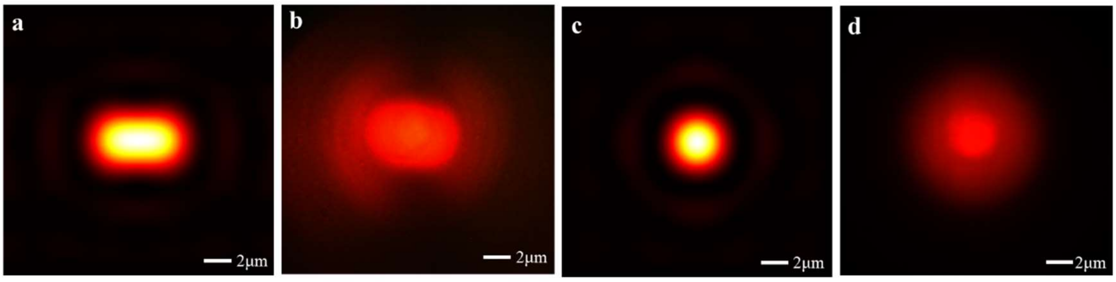

Supplementary Figure 15. The simulated and measured focal spots distribution. The **a** simulated and **b** measured focal spot distribution of the proposed a-DIC metasurface. The **c** simulated and **d** measured focal spot distribution of the proposed i-DIC metasurface.

Supplementary Note 9. Theoretical analysis of the edge detection effect for amplitude samples

The object complex amplitude transmission function can be written as $O(x,y)=a(x,y)e^{ib(x,y)}$ ($O(r,\theta)=m(r,\theta)e^{in(r,\theta)}$ in polar coordinate), where $a(x,y)$, $b(x,y)$, $m(r,\theta)$ and $m(r,\theta)$ are real functions. For an amplitude sample, the transmission function can be expressed as $O(x,y)=a(x,y)$ ($O(r,\theta)=m(r,\theta)$ in the polar coordinate system). Assuming that the introduced shear is $2\Delta s$, and the introduced bias retardation is $2\Delta\varphi$, two linear polarizers at the input and output are aligned to 45° and -45°.

In a-DIC microscopy, for *x*- polarization, the ideal imaging result is $a\left(x+\Delta s,\, y\right)\exp\left(i\Delta\varphi\right)$. Correspondingly, for *y*-polarization, it is $b\left(x-\Delta s,\, y\right)\exp\left(-i\Delta\varphi\right)$. The intensity of the anisotropic DIC imaging is

$$I_{aniso}=\left|a(x-\Delta s,y)e^{-i\Delta\varphi}\text{-}a(x+\Delta s,y)e^{i\Delta\varphi}\right|^2 \tag{38}$$

If the introduced bias retardation $2\Delta\varphi = 0$,

$$\begin{aligned} I_{aniso}&=\left|a(x-\Delta s,y)\text{-}a(x+\Delta s,y)\right|^2\\ &\approx 2\Delta s\left|\frac{\partial a(x,y)}{\partial x}\right|^2 \end{aligned} \tag{39}$$

So $I_{aniso}\propto\left|\frac{\partial a(x,y)}{\partial x}\right|^2$, here $\frac{\partial a(x,y)}{\partial x}$ represents the first-order differentiation of the image along the *x*-direction, which performs the results of anisotropic edge detection effect for the amplitude sample.

In i-DIC microscopy, for *x*- polarization, the ideal imaging result is $m\left(r+\Delta s,\theta\right)\exp\left(i\Delta\varphi\right)$. Correspondingly, for *y*- polarization, it is $m\left(r-\Delta s,\theta\right)\exp\left(-i\Delta\varphi\right)$. The intensity of the anisotropic DIC imaging is

$$I_{iso}=\left|m(r-\Delta s,\theta)e^{-i\Delta\varphi}\text{-}m(r+\Delta s,\theta)e^{i\Delta\varphi}\right|^2 \tag{40}$$

If the bias retardation $2\Delta\varphi = 0$,

$$
\begin{aligned}
I_{iso} &= \left| m(r-\Delta s,\theta)\text{-}m(r+\Delta s,\theta) \right|^2 \\
&\approx 2\Delta s \left| \frac{\partial m(r,\theta)}{\partial r} \right|^2
\end{aligned} \tag{41}
$$

So $I_{iso} \propto \left| \frac{\partial m(r,\theta)}{\partial r} \right|^2$, here $\frac{\partial m(r,\theta)}{\partial r}$ represents the differentiation of the image along *r*-direction, hence realizing the results of isotropic edge detection effect for amplitude sample.

Supplementary Note 10. The imaging results of the icons, numbers and letters

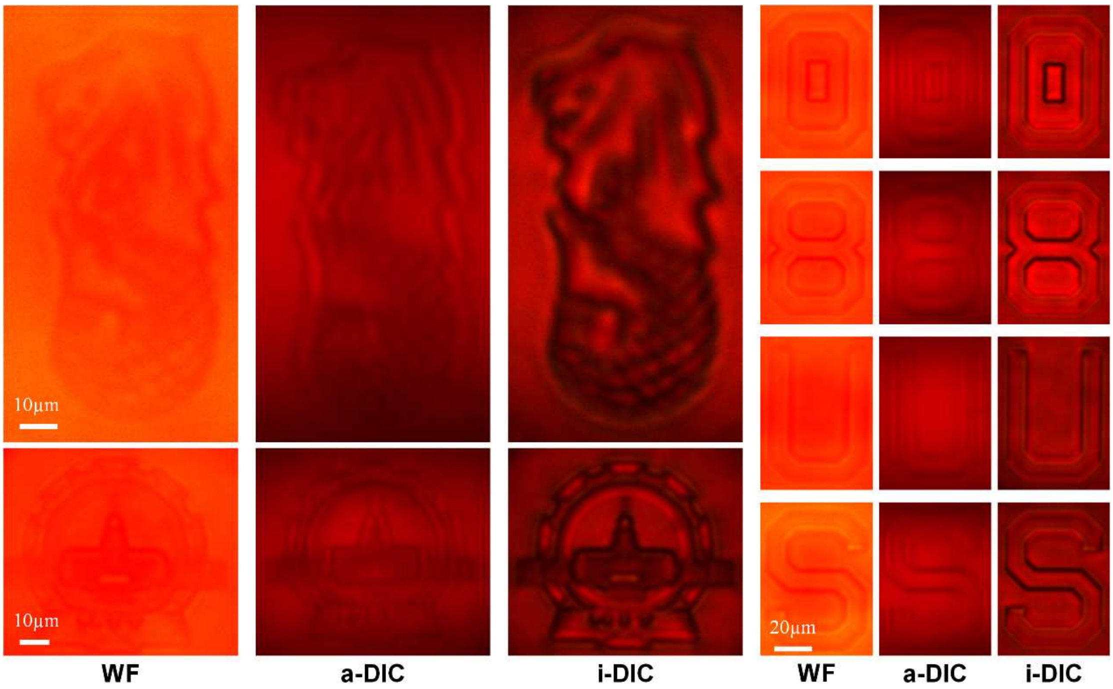

Supplementary Figure 16. The imaging results of the icons, numbers, and letters in widefield microscopy ('WF'), anisotropic DIC microscopy ('a-DIC'), and isotropic DIC microscopy ('i-DIC'). The diameter D = 200 μm, focal length f = 1mm, giving NA = 0.1. In anisotropic DIC metasurface, the shear $2\Delta s = 1$ μm and the bias $2\Delta\varphi = 0$. In isotropic DIC metasurface, the shear $2\Delta s = 1$ μm and the bias $2\Delta\varphi = \pi$.

Supplementary Note 11. The conventional DIC imaging results of the icons

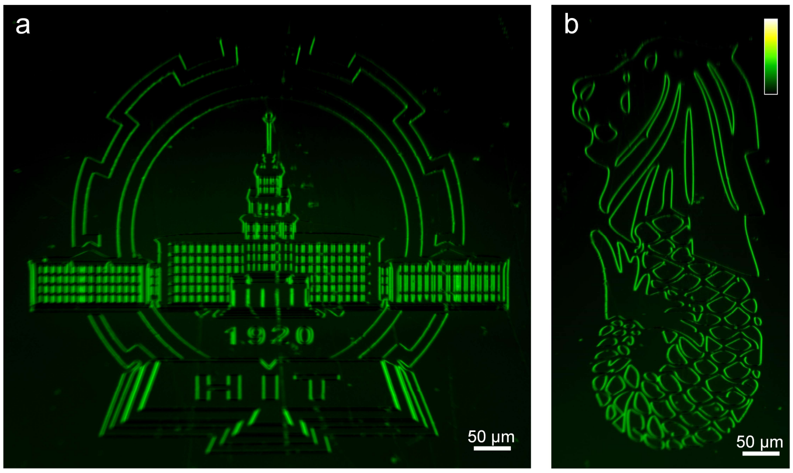

Supplementary Figure 17. The conventional DIC imaging results of the **a** HIT logo and **b** Merlion icon with NA = 0.15.

Supplementary Note 12. Image entropy

Entropy is an indicator of disorder or randomness, hence a measure of uncertainty. It can be considered as an assessment criterion for the amount of information. The higher the entropy, the more the information.

For a grayscale image, one intuitive approach is to consider the image as a matrix of pixels and compute the entropy using the following equation[5]:

$$H = -\sum_{k} p_k \log_2 p_k \tag{42}$$

where k is the number of gray levels and p_k is the probability associated with gray level k. To simplify it, let's consider a single-channel 8-bit image (256 intensity levels), then we can compute the p_k value as follows:

$$p_k = \frac{\text{Number of occurrences of the intensity level } k}{\text{Number of intensity levels}} \tag{43}$$

Reference

1. Yu, N. *et al.* Light Propagation with Phase Discontinuities: Generalized Laws of Reflection and Refraction. *Science* **334**, 333–337 (2011).
2. Goodman, J. W. *Introduction to Fourier Optics*. (W.H. Freeman, 2017).
3. Gu, M. *Advanced Optical Imaging Theory*. vol. 75 (Springer Berlin Heidelberg, 2000).
4. Preza, C., Snyder, D. L. & Conchello, J.-A. Theoretical development and experimental evaluation of imaging models for differential-interference-contrast microscopy. *J. Opt. Soc. Am. A* **16**, 2185–2199 (1999).
5. Gonzalez, R. & Woods, R. *Digital Image Processing*. (Pearson, 2017).
