# Supplementary material for: Single-shot isotropic differential interference contrast microscopy
Source: Nat Commun. 2023 Apr 12;14:2063. doi: 10.1038/s41467-023-37606-6 (PMC10097662; doi:10.1038/s41467-023-37606-6)
Supplement: Supplementary file 2 — Description of Additional Supplementary Files [file 41467_2023_37606_MOESM2_ESM.pdf]

Description of Additional Supplementary Files

File Name: Supplementary Movie 1
Description: Moving SiO_2 microspheres observed by i-DIC microscopy.

File Name: Supplementary Movie 2
Description: Moving SiO_2 microspheres observed by a-DIC and i-DIC microscopy.
