# Supplementary material for: Single-shot isotropic differential interference contrast microscopy
Source: Nat Commun. 2023 Apr 12;14:2063. doi: 10.1038/s41467-023-37606-6 (PMC10097662; doi:10.1038/s41467-023-37606-6)
Supplement: Supplementary file 5 — Reporting Summary [file 41467_2023_37606_MOESM5_ESM.pdf]

nature portfolio

Corresponding author(s): Guangwei Hu

Last updated by author(s): Feb 1, 2023

Reporting Summary

Nature Portfolio wishes to improve the reproducibility of the work that we publish. This form provides structure for consistency and transparency in reporting. For further information on Nature Portfolio policies, see our Editorial Policies and the Editorial Policy Checklist.

Statistics

For all statistical analyses, confirm that the following items are present in the figure legend, table legend, main text, or Methods section.

n/a	Confirmed	
[ ]	[x]	The exact sample size (*n*) for each experimental group/condition, given as a discrete number and unit of measurement
[ ]	[x]	A statement on whether measurements were taken from distinct samples or whether the same sample was measured repeatedly
[x]	[ ]	The statistical test(s) used AND whether they are one- or two-sided<br>*Only common tests should be described solely by name; describe more complex techniques in the Methods section.*
[ ]	[x]	A description of all covariates tested
[ ]	[x]	A description of any assumptions or corrections, such as tests of normality and adjustment for multiple comparisons
[x]	[ ]	A full description of the statistical parameters including central tendency (e.g. means) or other basic estimates (e.g. regression coefficient) AND variation (e.g. standard deviation) or associated estimates of uncertainty (e.g. confidence intervals)
[x]	[ ]	For null hypothesis testing, the test statistic (e.g. *F*, *t*, *r*) with confidence intervals, effect sizes, degrees of freedom and *P* value noted<br>*Give P values as exact values whenever suitable.*
[x]	[ ]	For Bayesian analysis, information on the choice of priors and Markov chain Monte Carlo settings
[x]	[ ]	For hierarchical and complex designs, identification of the appropriate level for tests and full reporting of outcomes
[x]	[ ]	Estimates of effect sizes (e.g. Cohen's *d*, Pearson's *r*), indicating how they were calculated

Our web collection on statistics for biologists contains articles on many of the points above.

Software and code

Policy information about availability of computer code

Data collection	No collected data, all data were captured by our own metasurface-based isotropic or anisotropic differential interference contrast(DIC) Microscopy, in which the details can be found in the Methods section.
Data analysis	All the Figures and dates were prepared and processed with CST Microwave Studio 2020, ImageJ v1.53g, Microsoft Visio 2016, 3D Max 2016, and Microsoft PowerPoint 2016, and videos were all produced by ThorCam 3.7.0 software and and processed by ImageJ v1.53g.

For manuscripts utilizing custom algorithms or software that are central to the research but not yet described in published literature, software must be made available to editors and reviewers. We strongly encourage code deposition in a community repository (e.g. GitHub). See the Nature Portfolio guidelines for submitting code & software for further information.

Data

Policy information about availability of data

All manuscripts must include a data availability statement. This statement should provide the following information, where applicable:
- Accession codes, unique identifiers, or web links for publicly available datasets
- A description of any restrictions on data availability
- For clinical datasets or third party data, please ensure that the statement adheres to our policy

All data generated and analyzed are included in the paper and its supplementary information, and another relevant data that support the findings of this study are also available from the corresponding authors upon reasonable request.

Human research participants

Policy information about studies involving human research participants and Sex and Gender in Research.

Reporting on sex and gender	Breast cancer is a common endocrine system tumor in women, and its incidence in male patients is very low. Therefore, all samples were collected from female patients.
Population characteristics	Two Asian women with breast cancer, aged 60 and 48, were enrolled in this study.
Recruitment	This is not relevant to our study.
Ethics oversight	The study followed the tenets of the Declaration of Helsinki and was approved by the medical ethics committee of the Third Affiliated Hospital of Harbin Medical University. All patient samples were obtained after informed consent was provided.

Note that full information on the approval of the study protocol must also be provided in the manuscript.

Field-specific reporting

Please select the one below that is the best fit for your research. If you are not sure, read the appropriate sections before making your selection.

[x] Life sciences [ ] Behavioural & social sciences [ ] Ecological, evolutionary & environmental sciences

For a reference copy of the document with all sections, see nature.com/documents/nr-reporting-summary-flat.pdf

Life sciences study design

All studies must disclose on these points even when the disclosure is negative.

Sample size	The technology has been validated with several tissues and cells for the structural and functional imaging. No sample size calculation was performed. But for each experiment with specific aims, the same imaging protocols were repeated with more than 4 times, which are sufficient for technical demonstration of our method.
Data exclusions	No data was excluded from the analysis.
Replication	All imaging experiments were repeated with more than 4 times and could be repeated each time. To show the imaging performance, representative imaging results were included in the manuscript.
Randomization	Experiment tissues, cells and imaging region of interests were randomly chosen without prior bias, and data analysis was performed using the same method.
Blinding	This is not relevant to our study because the main result of our work is a new type of imaging technique. The imaging technique is well understood physically and its performance was demonstrated by showing representative imaging results

Reporting for specific materials, systems and methods

We require information from authors about some types of materials, experimental systems and methods used in many studies. Here, indicate whether each material, system or method listed is relevant to your study. If you are not sure if a list item applies to your research, read the appropriate section before selecting a response.

Materials & experimental systems

n/a	Involved in the study	
x		Antibodies
	x	Eukaryotic cell lines
x		Palaeontology and archaeology
x		Animals and other organisms
x		Clinical data
x		Dual use research of concern

Methods

n/a	Involved in the study	
x		ChIP-seq
x		Flow cytometry
x		MRI-based neuroimaging

Eukaryotic cell lines

Policy information about cell lines and Sex and Gender in Research

Cell line source(s)	The cells came from the Chinese Academy of Sciences. The cells and tissue were taken from female samples.
Authentication	According to the requirements of the experiment, only morphological observation was performed in this experiment, and verification of relevant biological functions was not required. Therefore, relevant verification of cell lines was not involved here.
Mycoplasma contamination	The cell lines were not tested for mycoplasma contamination.
Commonly misidentified lines (See ICLAC register)	Not applicable.
